# “Children are precious cargo; we don’t let them take any risks!”: Hearing from adults on safety and risk in children’s active play in schools: a systematic review

**DOI:** 10.1186/s12966-022-01344-7

**Published:** 2022-09-01

**Authors:** Alethea Jerebine, Katie Fitton-Davies, Natalie Lander, Emma L. J. Eyre, Michael J. Duncan, Lisa M. Barnett

**Affiliations:** 1 School of Health and Social Development, Faculty of Health, Deakin University, Geelong, VIC Australia; 2 Centre for Sport, Exercise and Life Sciences, Coventry University, Coventry, UK; 3 Research Institute for Sport and Exercise Sciences, Liverpool John Moores University, Liverpool, UK; 4 Institute for Physical Activity and Nutrition, School of Exercise and Nutrition Sciences, Deakin University, Geelong, VIC Australia; 5 Institute for Physical Activity and Nutrition, Deakin University, Geelong, VIC Australia

**Keywords:** Risky play, Physical activity, Recess, Socio-ecological model, Qualitative, Risk tolerance

## Abstract

**Background:**

Understanding determinants of children’s outdoor play is important for improving low physical activity levels, and schools are a key setting for both. Safety concerns shape children’s opportunity to play actively outdoors, therefore, this qualitative evidence synthesis aimed to i) examine adult (e.g., parent, teacher, yard supervisor, principal) perspectives on safety and risk in children’s active play during recess in elementary and/or middle schools, and ii) identify how safety and risk influence playground supervision and decision making in this setting.

**Methods:**

Six electronic databases were systematically searched in March 2021, with an updated search in June 2022. Records were screened against eligibility criteria using Covidence software, and data extraction and synthesis were performed using predesigned coding forms in Microsoft Excel and NVivo. Framework synthesis methodology was employed, guided by a conceptual framework structured on the socio-ecological model (SEM) and affordance theory.

**Results:**

From 10,370 records, 25 studies were included that represented 608 adults across 89 schools from nine countries. The synthesis identified 10 constraining and four affording factors that influenced whether school staff were risk-averse or risk tolerant during recess, and, in turn, the degree to which children’s play was managed. Constraining factors stemmed from fears for children’s physical safety, and fear of blame and liability in the event of playground injury, which shaped parent, school staff and institutional responses to risk. Interrelated factors across SEM levels combined to drive risk-averse decision making and constraining supervision. Emerging evidence suggests children’s active play in schools can be promoted by fostering a risk tolerant and play friendly culture in schools through play facilitation training (e.g., risk-reframing, conflict resolution) and engaging stakeholders in the development of school policies and rules that balance benefits of play against potential risks.

**Conclusions:**

Findings show several socio-cultural factors limited the ability of school staff to genuinely promote active play. Future work should seek to foster risk tolerance in schools, challenge the cultural norms that shape parent attitudes and institutional responses to risk in children’s play, and explore novel methods for overcoming policy barriers and fear of liability in schools.

**Trial registration:**

PROSPERO registration: CRD42021238719.

**Supplementary Information:**

The online version contains supplementary material available at 10.1186/s12966-022-01344-7.

## Introduction

Physical activity (PA) is an important health behaviour with implications for many developmental outcomes in children including musculoskeletal development, cardiovascular health, and mental wellbeing [1–3]. Moreover, evidence of positive associations between PA and cognitive function, as well as academic achievement, has grown [4–6]. Play is a key domain of children’s PA [7], with recognised developmental and wellbeing benefits in its own right [8–11]. Play is also acknowledged as a fundamental human right, enshrined in article 31 of the Convention on the Rights of the Child (CRC) [12]. Children’s PA during play is commonly referred to as active play [13, 14], although variations abound and a consensus definition is lacking [15]. This review adopts the definition proposed by Truelove and colleagues: “*active play is a form of gross motor or total body movement in which children exert energy in a freely chosen, fun, and unstructured manner*” ([13], p.164).

Active play provides significant potential for increasing children’s PA levels [7], which remain persistently low and may be declining in some countries [16–18]. Schools offer a valuable opportunity to promote active play as children spend large portions of their day at school, usually with dedicated free play periods spent in the school yard between formal academic lessons [19, 20]. Though schools are the most researched of children’s PA settings [21], systematic reviews of recess interventions have reported inconsistent effects on PA levels [22, 23]. This suggests the range of active play determinants in schools, particularly the interaction between individual children (their age, gender, and interests) and wider influences in the school system, require greater understanding. A relatively new area of research focuses on the role of risk-taking and challenge in children’s active play [24, 25], and the potential impact adult safety concerns and risk aversion have on the social and physical play environment children experience [26, 27].

Although attitudes to play and safety vary across countries, cultural patterns have emerged in developed nations, including a protective parenting mindset, and bureaucratic and risk averse public health and safety policies and legislation [28, 29]. Researchers contend these forces are evident in the declining opportunities children have for play outdoors [30, 31] and increasing monitoring and surveillance children experience [9]. Moreover, the development of safety regulations for children’s play environments and standardised symmetrical playgrounds engineered to reduce injury and liability risk, further illustrate the issue [32–35]. The typical ‘KFC‘ playground, containing a **K**it of prefabricated play equipment, a **F**ence, and a **C**arpet of rubber safety surfacing, is a familiar symbol of this shift [36, 37]. Alongside these changes, an increasing interest in the concept of ‘risky play’ has emerged, which aims to articulate its significance for healthy child development [38, 39], and potential for improving children’s PA levels [24]. Defined as “*thrilling and exciting forms of physical play that involve uncertainty and a risk of physical injury*” ([40], p.22), advocates contend risk-taking in play is a natural and necessary part of active play, where children push physical and psychological boundaries, practice new skills, and experience the satisfaction and joy of mastery [38, 41, 42]. A range of risky play categories have been identified, including play at great heights, play at high speeds, and rough and tumble play [43]. Importantly, what constitutes risky play, e.g. what height becomes a ‘great height’, is subjective and will vary relative to a child’s size, physical literacy, and other characteristics [44, 45]. Gibson’s theory of affordances provides a useful basis for examining how children interact with their environment and the play opportunities they are ‘afforded’ [46]. In the context of risky or active play, affordances are the opportunities children have to run, jump, climb, swing, balance, chase and wrestle etc. [47]. In a school setting, play affordances, and the degree of risk and challenge afforded in the playground, will vary across the student population.

Like PA, active play is a multidimensional behaviour influenced by a range of individual, environmental, and socio-cultural factors [16, 48]. Ecological models, such as Bronfenbrenner’s socio-ecological model (SEM), offer a useful lens of analysis for conceptualising this complex array of determinants [49]. The fundamental principle of the SEM is that there are multiple interacting levels of influence on health behaviours (like active play), including intrapersonal factors (biological, psychological), interpersonal, physical environment, institutional, policy and societal factors [50]. In a school setting, adults are often the ‘gatekeepers’ of children’s active play, and their decision making plays a significant role in the movement opportunities children are afforded. Moreover, schools carry a responsibility for the welfare of large numbers of children in their care, which influences decision-making at various ecological levels [51, 52]. Therefore, understanding adults’ perspectives on safety and risk in children’s active play during recess, may help inform how this issue is perceived, and provide insight into the forces that shape children’s play affordances in this complex setting [53, 54].

Research examining children’s perspectives on recess [55] and safety and risk in active play in schools [56] has been subjected to systematic review, however, to our knowledge, there has been less attention to adults’ perspectives and experiences. A previous review examined barriers and facilitators to adventurous play in schools, however, its scope was narrow with respect to play (focussing on “exciting, thrilling play where children are able to take age-appropriate risks” in contrast to active play more broadly, as defined by Truelove and colleagues and described above), and most included studies (six of nine) were evaluations of adventurous play interventions, which may have limited relevance outside of an intervention context ([57], p.21). Additionally, the scope of this previous review was wide with respect to children’s settings (including early childhood and special education settings in addition to schools), which may have generated insights specific to those settings [57]. Therefore, this qualitative systematic review aimed to examine adult (e.g., parent, teacher, yard supervisor, principal, administrator) perspectives on safety and risk in children’s active play during recess in elementary and/or middle schools. Through application of the SEM and affordance theory, the review aimed to identify how safety and risk shape decision making in schools and playground supervision during recess.

## Methods

This qualitative systematic review follows the Preferred Reporting Items for Systematic Reviews and Meta-Analyses (PRISMA) guidelines [58], and the Enhancing Transparency in Reporting the Synthesis of Qualitative Research (ENTREQ) statement [59] (Additional file 1), and was prospectively registered with PROSPERO (CRD42021238719). The review concept began as a synthesis of qualitative research conducted with both adults and children, and the methods described below reflect this. However, as the review progressed, it became apparent the data generated through research with adults was sufficiently rich to warrant its own review. Thus, this systematic review has synthesised research conducted with *adults* to generate insights into their perspectives, experiences, and behaviour regarding safety and risk in children’s active play during recess.

### Literature search strategy

Six electronic databases were systematically searched to identify relevant studies: Education Source, ERIC, MEDLINE, PsycINFO, SPORTDiscus, and Embase. A search strategy, which combined terms for ‘teacher’, ‘principal, ‘parent’, ‘child’, ‘school’, ‘active play’, and ‘recess’, was developed and adapted for each database. The search was restricted to articles published from 2000 onwards to concentrate on contemporary perspectives on children’s active play in schools. The original search was performed in March 2021 and updated in June 2022. Reference lists of included studies and literature reviews identified during the development of the initial conceptual framework (See Additional file 3) were hand-searched for additional articles. A comprehensive description of the search strategy is provided in Additional file 2.

### Study screening and selection

**Fig. 1:**
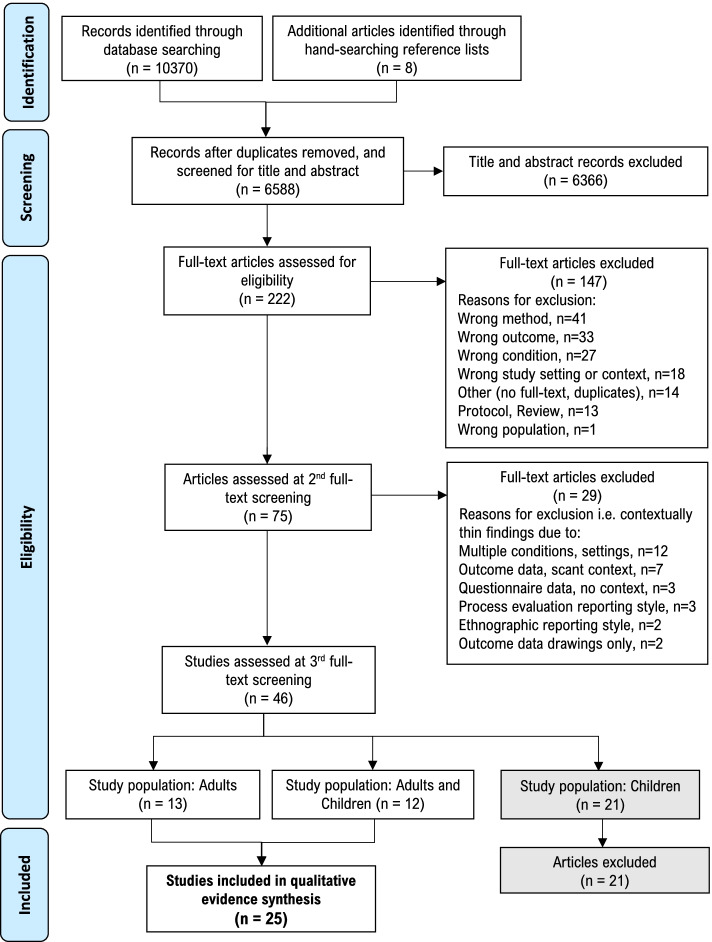
PRISMA flowchart

Search results were imported into Clarivate Analytics EndNote X9, where duplicates were removed, before remaining records were imported into Covidence [60] for screening against predefined eligibility criteria as described below and in Table 1. Study screening and selection was undertaken in four stages. For the first three stages, records and/or articles were screened for eligibility independently by teams of two reviewers (AJ, EE, KF, LB, NL) using predefined screening tools and discrepancies were discussed among the wider team until consensus was reached. For **Stage 1** title abstract screening, studies were required to meet six eligibility criteria: article type, population, setting, context, condition, and research method. In **Stage 2** full-text screening, an additional ‘risk or safety outcome’ criterion was applied for inclusion in the review. However, upon commencement of data extraction, it became apparent that some studies lacked sufficient detail to make a meaningful contribution to the aims of this review. For example, a study may have mentioned that ‘safety’ was a concern for a teacher, without explaining what the safety concern related to. As such, a further screening stage (**Stage 3**) was introduced to identify and exclude any studies that lacked ‘contextually thick data’ (defined in Table 1) [54]. Reasons for excluding articles at both stage 2 and 3 are reported in Fig. 1. The number of studies meeting the eligibility criteria at the end of stage 3 (*n* = 46), and the depth of data produced, led to a further stage of screening to narrow the scope of the review. For the final, **Stage 4 **screening, the study population was narrowed to adults, with research conducted with children subjected to a separate review [56].

**Table 1:** Eligibility criteria for inclusion of studies in the framework synthesis

	Inclusion	Exclusion
**Stage 1: Title/ Abstract Screening**		
Article type	Original research published in peer-reviewed academic journals.	Conceptual or theoretical papers, opinion pieces, reviews.
Population	Typically developing children or early adolescents with a mean age between 5 and 14 years. AND/OR Adults with a role relevant to children in the school setting (e.g. teachers, yard duty supervisors, principals, school administrators, school nurses, parents). The aim of the research must be to explore adults’ behaviour and/or perceptions in relation to children’s active play and/or risky play in schools.	Children older or younger than the age range specified. Children with a medically diagnosed condition e.g. asthma, autism, epilepsy, intellectual disability, immune disorder etc. Adults’ perceptions of PE, active lessons, structured recess or children’s active play or risky play outside of school.
Study setting	Elementary or middle school (or equivalent) settings	Before- or after-school programs, early childhood programs, high schools.
Context	Recess: defined as “*the non-curriculum time allocated by schools between lessons for youth to engage in leisure activities*” ([61], p.3).	Structured classroom activity breaks, active lessons, physical education classes, outdoor education programs, outdoor learning.
Condition	Active play or risky play: Active play: defined as *“a form of gross motor or total body movement in which children exert energy in a freely chosen, fun, and unstructured manner”* ([13], p.164). Risky play: defined as *“thrilling and exciting forms of play that involve uncertainty and a risk of physical injury”* ([40], p.22). In recognition of the wide variation in the literature for terms pertaining to children’s play, the following alternative terms were included: outdoor play, free play, unstructured play, physical activity during play, unstructured physical activity, child play, challenging play, and adventurous play.	Structured-play, structured-recess programs such as walking interventions, teacher-organised recess activities.
Research method	Original research employing at least one qualitative research method such as focus groups, observation, or walking interviews. Mixed methods studies were included if data from the qualitative components could be extracted and analysed independently of the quantitative results.	Quantitative research methods e.g. experimental, quasi-experimental, cross-sectional and cohort studies.
**Stage 2: Full-Text Screening**		
Risk or safety outcome	Safety or risk-related findings or themes in relation to children’s active play and/or risky play. Risk: defined as *“the effect of uncertainty (whether positive or negative) on objectives”* [62]. Safety: defined as *“a state in which hazards and conditions leading to physical, psychological or material harm are controlled in order to preserve the health and well-being of individuals and the community”* [63]. Notably this definition includes both physical and psychological safety.	Study findings relating to safety and risk in schools that is not directly related to active play or risky play, such as: gun violence, soil or air pollution, microbial infections.
**Stage 3: Full-Text Screening**		
Outcome data is contextually thick	Risk and safety findings must be contextually *thick*. Contextually thick descriptions identify both an ‘issue’ (e.g. a risk or safety finding in play) and its context, and the context provides the social or cultural meaning to the issue, thereby aiding it’s symbolic importance and understanding [54].	Risk or safety findings are contextually *thin*, due to: 1. Scope: multiple conditions or setting domains investigated; 2. Outcome data reported too brief; 3. Method: Questionnaire within insufficient qualitative data; 4. Process evaluation reporting of intervention or outcomes with thinly described data; 5. Ethnographic reporting method where ‘findings’ cannot be differentiated from the remainder of the article; 6. Method: relevant data limited to children’s drawings without children’s own description of meaning
**Stage 4: Full-Text Screening**		
Population: adults	Adults with a role relevant to children in the school setting (e.g. teachers, yard duty supervisors, principals, school administrators, parents). Studies where both children and adults were participants were included if data relating to adult participants could be extracted and analysed independently of the child participants.	Children or early adolescents only

### Synthesis method

The framework synthesis method was selected, which is a systematic but flexible approach to studying the complexity intrinsic to health and social sciences research [64, 65]. Framework synthesis comprises two stages and five overlapping steps [66] as detailed in Table 2.

**Table 2:** Framework synthesis method applied in the current review

Framework synthesis stage	Synthesis steps	Application in this review
***Stage 1*** *Developing an initial conceptual framework*	1. **Familiarisation**: Becoming immersed in the data	Undertaken during full-text screening and study selection (both stages), in addition to reading quantitative literature, systematic and narrative reviews for the field, and handsearching references.
	2. **Framework selection**: Identification of either an existing framework or key theory and themes in the literature to inform the framework	Systematic extraction of salient themes and findings from 18 studies identified in Step 1, identification of relevant theory and definitions (see Additional file 3 for full description).
***Stage 2*** *Recognising patterns of data through an iterative process of aggregation and configuration*	3. **Indexing**: Systematically tagging and labelling key themes in the data	Data extracted, labelled, and indexed in NVivo software, using codebook developed from initial conceptual framework. Data not fitting framework analysed inductively.
	4. **Charting**: Devising a series of thematic charts to allow the full pattern across papers to be explored and reviewed	Themes developed and revised iteratively in NVivo. Findings/ themes charted in Excel, patterns across data and studies explored.
	5. **Mapping and interpretation**: Drawing together the synthesis, consideration of how the themes answer the review question	Conceptual framework developed further to reflect review findings. Analysis of relationships between themes, themes mapped and illustrated in Figures using PowerPoint.

The ‘Framework synthesis stage’ and ‘Synthesis steps’ columns are informed by the work of Brunton et al. [64] and Gough et al. [66]

### Initial conceptual framework and codebook development

The development of an initial conceptual framework to guide the synthesis, and the evolution of the framework as the review progresses, are the defining features of framework synthesis [66]. In the current review, the initial conceptual framework was systematically developed from the literature as no existing framework could be identified. A supporting codebook to guide data extraction was subsequently developed. A description of the development process, and the resultant framework and codebook, are summarised in Table 2 and provided in Additional file 3. The framework was structured on the SEM [49] and Gibson’s theory of affordances [46]. The initial conceptual framework represented five levels of influence on children’s active play during recess (individual, interpersonal, physical environment, institutions, policy, and society), and 25 risk and safety themes, which may ‘afford’ or ‘constrain’ active play in schools.

### Data collection

Study characteristics were independently extracted by two authors (AJ, KF) using a standardised data extraction Excel spreadsheet, developed by the primary author. Data extracted included: date, country, discipline, researcher aims, study design, theoretical framework, sampling methods, school and participant characteristics, data collection and analysis techniques, and rigour. Any discrepancies in extraction were discussed until consensus was reached.

The methodological quality of each study was evaluated using the Critical Appraisal Skills Programme Qualitative Checklist (CASP Checklist) [67, 68]. In recognition of the diversity in qualitative research approaches and reporting styles, which can influence appraisal outcomes, no study was excluded based on appraisal results [68, 69]. The CASP Checklist consists of two screening questions (relating to study aims and appropriateness of qualitative methodology to those aims) and eight appraisal questions (research design, recruitment strategy, data collection, reflexivity, ethical issues, rigour of data analysis, and the reporting and value of findings) [67]. All eligible studies were independently appraised by two authors (AJ, MD) using Covidence software. The CASP Checklist was modified to include ‘Somewhat’, where an item was partially met, in addition to the ‘Yes’ (totally met) ‘No’ (not met) and ‘Can’t tell’ (not enough information to make a judgement) options; consistent with recently published approaches [70]. AJ and MD developed and applied criteria for what constituted each answer option for the 10 CASP items. Any disagreements in appraisal were discussed and consensus reached.

A challenge in qualitative evidence synthesis is deciding what constitutes the findings of primary studies [66]. For this review, anything labelled ‘results’ or ‘findings’, were taken to be study results and consisted either of verbatim quotations from study participants or findings and observations reported by authors [66]. Observations, author interpretations and quotations were given equal weighting. For the studies that included both child and adult participants, only data relating to the adult participants were extracted. For extraction of findings, studies were imported into QSR NVivo software (version 1.5) to aid data management and analysis.

### Analysis and synthesis of results

Data identified as risk or safety findings were extracted, labelled, and indexed (coded) by one author (AJ) using the codebook (Additional file 3). Prior to indexing, the codebook was piloted by the author team (AJ, EE, LB, NL) with a subset of four studies (16%) to enhance trustworthiness of the synthesis. Coding between authors for each study were compared and discussed and the codebook was refined. As detailed in Table 2, extracted data were first labelled descriptively (indexed) and then analysed both deductively (using the codebook) and inductively (e.g. where extracted data did not translate into pre-existing themes), to develop new themes, consistent with thematic analysis [66, 71]. Findings were then charted, mapped, and interpreted to identify patterns across data and studies, through a process of configuration [66]. This was an iterative process, whereby, themes evolved as more data were synthesised, resulting in an emergent framework, which integrated the initial conceptual framework with the new concepts and themes [64].

### Positionality and reflexivity

In the interest of trustworthiness and transparency, it is important researchers provide context for their work, such as professional background and worldview [72]. The current review adopts a critical realist perspective, which proposes that knowledge is a social product, and our knowledge of reality is shaped by our perceptions and beliefs [73]. Authors in this review have professional backgrounds and expertise in education (EE, KF, NL), health promotion (AJ, LB), physical literacy (AJ, EE, KF, LB, NL, MD), public health (AJ, LB), qualitative research methods (AJ, EE, KF, LB, NL), sport science and motor skill development (EE, KF, LB, MD, NL), and systematic reviews (AJ, EE, LB, MD, NL). The authors acknowledge the influence these backgrounds had on development of the review question, study design and evidence synthesis. Throughout the review process, the authors met frequently to discuss team reflections, including discussions about how safety and risk were perceived and reported in the literature, what constituted a safety or risk finding, and the influence of differing ontological or epistemological perspectives [74].

## Results

### Study selection

A total of 9664 records were identified in the original database search in March 2021, an additional 706 records were identified in the updated June 2022 search, and eight articles were identified through manual searching. Following four stages of detailed screening (see Table 1), 25 studies were identified for inclusion in the framework synthesis. The screening process is illustrated in a PRISMA flow diagram in Fig. 1, which sets out the pre-defined reasons studies were excluded at the 1st and 2nd full-text screening stages, and the split between adult and child-based research at the final stage.

### Characteristics of included studies

Characteristics of included studies are described in Additional file 4. Of the 25 studies, six were conducted in the USA [75–80], five in England [81–85], and four studies in both Australia and Canada [86–89]. Two studies were conducted in Turkey [90, 91] and one in each of Iceland [92], New Zealand [93] and Sweden [94]. While one study was conducted in both Sweden and France [95].

Not all studies clearly specified the number of participants, particularly where school playground observation was used, however, of reported data, at least 608 adults across 89 schools participated. Of participants, 76 were principals or school administrators, 244 were teachers, 30 were playground supervisors, and 100 were other adults in the school system, including school nurses, and playworkers (practitioners who work with students to promote play through teaching recess games, introducing conflict resolution tools, and encouraging positive language and inclusive behavior [80]). Additionally, 147 participants were parents. Other participant characteristics, including gender, qualifications, and experience, were inconsistently reported. One study was conducted in a middle school [76], and the remaining were conducted in elementary schools or the international equivalent (with children’s ages ranging from 4 to 12 years), except for one study, which included participants from both elementary and middle schools (with children aged up to 14 years) [88]. The school setting was also inconsistently or not reported, however, we identified 12 studies were conducted in urban schools [77, 79–82, 88, 92–98], one study in a rural school [89], and four studies in both urban and rural schools [83–85, 95].

Although not consistently reported, a range of study designs and methodologies were employed, including participatory action research (*n* = 3) [75, 89, 92], ethnography (*n* = 3) [81, 84, 85], formative, process, or outcome evaluations (*n* = 3) [78, 93, 96], phenomenology (*n* = 3) [77, 90, 91], qualitative descriptive (*n* = 2) [87, 99], explorative (*n* = 2) [76, 95], case study (*n* = 2) [86, 88], and field study (*n* = 1) [94] designs. The most common method for eliciting participant perspectives were interviews (*n* = 17) [76–78, 80, 81, 83–86, 90–93, 95–97, 99], while four studies conducted focus groups [75, 88, 89, 94], and four used questionnaires [79, 82, 87, 98]. Additionally, over half the studies (*n* = 14) employed more than one data collection method, such as playground observations in combination with interviews or focus groups. A range of analysis techniques were employed; of named methods, content analysis was most common (*n* = 5) [77, 90, 91, 93, 94], followed by thematic analysis (*n* = 4) [82, 88, 92, 95], constant comparative analysis (*n* = 3) [79, 96, 98], framework analysis (*n* = 2) [86, 89], ethnographic analysis (*n* = 2) [84, 85], and hermeneutic interpretive analysis (*n* = 1) [97].

### Quality appraisal

The quality appraisal of included studies is provided in Additional file 5. In summary, almost all studies clearly stated the research aims and provided a study design appropriate to achieving these aims. A clear statement of research findings and a discussion of the implications of the research were also well reported by most studies. Research methods were reported inconsistently, particularly, descriptions of ethical considerations, the consent process, and recruitment methods, were lacking. Additionally, in 76% of studies, the relationship between the researcher and participants was not critically examined, nor were the researchers’ positionality, and the potential for bias during the research process, discussed.

### Risk and safety themes

**Fig. 2:**
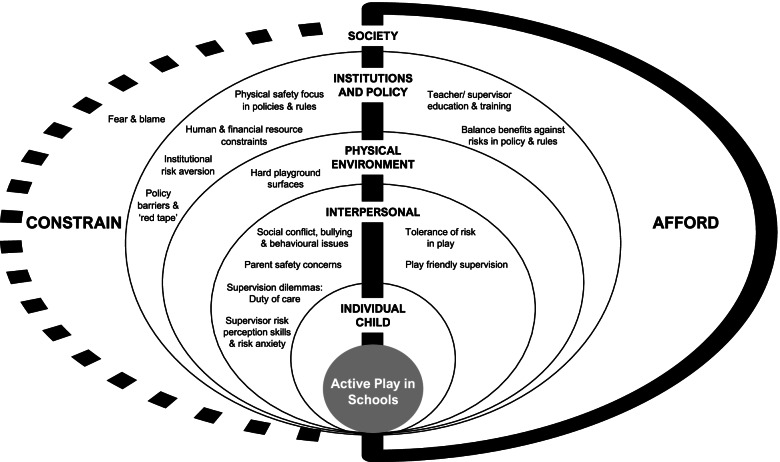
Socio-ecological model of risk and safety factors that shape children’s active play in schools. Legend: The socio-ecological model represents the emergent conceptual framework for risk and safety factors that shape children’s active play in schools from the perspective of adults. The framework consists of 10 constraining factors and 4 affording factors across four levels of the SEM: Society, policy and institutions, physical environment and interpersonal

Fourteen risk and safety themes were identified in the synthesis. The themes are depicted in the emergent conceptual framework (Fig. 2), which illustrates the interacting factors across four SEM levels that afford or constrain children’s active play during recess. The synthesis findings are presented below by SEM level, with affording and/or constraining influences described for each theme in the framework. Where participants are described, for example, as an ‘English teacher’ or ‘American principal’, we do not assert that this is their nationality or ethnicity, rather that they were a teacher or principal in the country where the study was conducted.

#### Individual child characteristics

No studies investigated adult perspectives on how children’s individual characteristics influenced their engagement with risk and ability to stay safe while playing actively in schools.

#### Interpersonal

##### Social conflict, bullying and behavioural issues

Social and behavioural issues during recess were a key concern for playground supervisors, teachers, and principals, and ranged from arguments, fights, or exclusion, through to bullying and sometimes physical violence [75–78, 80–83, 85, 87, 99]. Supervision staff commonly responded to behavioural issues by constraining play through reprimands (e.g., *“[Children] told to stand still on one spot until they learned to behave*” ([85], p.73), withdrawal of play affordances (e.g., equipment removed, children assigned to “the wall”) [77, 78, 82, 87] or expulsion from the playground (e.g., sent to the principal’s office) [83, 85, 87]. Yard supervisors in an American study reported they “*spend a lot of time resolving conflicts*” ([78], p.110). While an English teacher summed up her perspective on teachers’ core playground task as “*you’re solving problems*” ([81], p.255). An Australian principal explained his perspective on recess: “*I think bullying is an issue at every school… yes it will always be an issue. I think that while there is a zero-tolerance policy it happens behind the scenes*” ([99], p.275). Adults’ perceptions of the causes for these issues varied widely, including limited space, equipment, and things to do [75–77, 80, 85, 87, 93, 99], lack of structure in playground [77, 87], too many rules [83, 85], children not playing by the rules [78, 80], and children lacking conflict resolution skills [76–78, 80, 87]. As explained by an American middle school recess supervisor, “so [at our school, we] give the kids lots of things to do, because in the absence of anything to do, they’ll come up with some behavior we don’t want them to” ([76], p.4).

##### Parent safety concerns

The safety concerns of parents were commonly reported; however, this was largely limited to the perceptions and experiences of teachers, principals, and other supervising adults, as only five studies included parents as participants [77, 86, 88, 97, 98]. According to both parents and school staff, parental concerns for children’s safety in active play centred on physical injury [77, 83, 86, 89, 96, 98], potential illness (arising from wet or cold weather play) [93], and an associated issue of clothing damage or soiling [81, 83], all of which influenced children’s access to playground equipment and spaces (such as grassed fields), as well as which games and activities were permitted or forbidden. As an Australian teacher explained: “*Parents seem to be a lot more anxious about what can happen to their children. Parents have this fear that, you know that [children] are always at risk*” ([98], p.231). While an English head teacher explained: “*[we’re] under a lot of pressure from parents and … [we’re] currently under investigation from the LEA [Local Education Authority] because of a parental complaint about the way a child’s playground injury had been dealt with*” ([83], p.56). Two Australian studies provided some insight into the source of parent safety concerns, which included beliefs the world has become more dangerous [97], uncertainty in the face of an overwhelming amount of parenting information [98], and fear of negative evaluation by others [97, 98].

##### Supervision dilemmas: duty of care

School staff commonly described professional dilemmas as reasons for constraining children’s active play, primarily duty of care responsibility [78, 81–83, 96, 98, 100] and a fear of negative evaluation (by colleagues or parents), blame (by parents or superiors), or liability in the event of an injury occurring [83, 86–89, 96–98]. Duty of care responsibilities were perceived as “*challenging*” [78], “*overwhelming*” [81] and “*unfair*” [96], and participants were concerned they may lose their jobs [89, 96, 98] or be personally liable should a child be injured in play [83, 86–88, 96]. Participants in several studies identified ‘surveillance’ and ‘safety’ as the chief priority during recess [76, 78, 81, 82, 95, 96].) As a French principal explained, “no, we don’t play with them. It’s surveillance, only surveillance” ([95], p.145). While school staff in an English study believed there was a ‘correct’ use of the playground and “children need to be guided on how to play safely” ([82], p.13). In some schools, fear of repercussion for playground injuries or play perceived as unsafe, led to constraining supervision practices even when it conflicted with supervisors’ own beliefs about play safety [94, 96, 98]. An Australian teacher illustrated this: “*If it was my child, of course I’d let her do it [go down a slide head first]. But would I let someone else’s child? No! The risk goes up about 300%!”* ([98], p.231). In contrast, a Swedish teacher described being more restrictive with her own children than at school: “*I am a proper hen around my own children, but I don’t want the school children to live through that*” ([94], p.6).

##### Supervisor risk perception skills and risk anxiety

Lack of confidence or skill in assessing and managing risk and safety in the playground were commonly reported. School staff had difficulty distinguishing between dangerous activities and play with risk that children could safely assess and manage themselves [81, 84, 94, 96]. Likewise, some school staff found it difficult to distinguish between play fighting games and real conflict between children [84, 85, 89], or disapproved of rough and tumble play because they perceived it was, or could evolve into, aggressive behaviour [75, 76, 81, 83, 87, 89, 96]. As such, participants described intervening in play as a precautionary measure: because duty of care responsibilities were always front of mind and it was not always clear how great the injury risk was, the default behaviour was to constrain play [76, 78, 81–83, 94, 96, 98]. As an Australian teacher explained: “*Children are precious cargo! We don’t let them take any risks!*” ([98], p.231). Similarly, teachers and midday supervisors in an English study, took the approach of “*if in doubt, ban it!*” ([83], p.57). Some participants described a generalised anxiety about uncertainty and risk in play, and a fear of what might happen [83, 89, 96, 98]. An Australian teacher summed up her concerns by saying: “*something could happen to somebody – I think that’s a teacher’s natural instinct to be worried that something could happen*” ([96], p.40).

##### Tolerance of risk in play

In contrast, school staff and parents also expressed risk tolerance and positive attitudes towards children’s play, describing children’s ability to keep themselves safe while taking risks [86, 94, 97], and the importance of learning risk management skills through experience and adult support [89, 93, 94, 96, 97]. Some school staff perceived children have a natural inclination for risky play and allowing children to challenge themselves was valuable enough to outweigh the possible risk of minor injuries [86, 89, 92, 93]. Canadian teachers participating in a playground naturalisation project (schoolyard ‘greening’ through creating gardens, planting trees, manipulating topography [e.g., dirt mounds sown with grass] and adding natural materials like logs, rocks, and sand) explained how they accommodated this: “W*hen [permitting] climbing trees [for the first time], we have agreed on a height that won’t give us too many stressful thoughts*.” ([86], p.119). While for Icelandic teachers, playing with sticks was acceptable and encouraged, as a teacher said: “*One stick can be like gold to them*” ([92], p.160). School staff explained how building an appreciation of the benefits for children helped improve risk tolerance. For example, risk-reframing workshops helped Australian teachers examine their supervision practices in the context of their desired outcomes for children, and how they might address their own anxieties about uncertainty and reframe risk as an opportunity for learning and development [97, 98]. Additionally, schools reported that focusing on shared values and desired outcomes for children, such as confidence, problem-solving skills, and resilience, also helped generate the support of parents in addition to improving teacher’s risk tolerance [76, 86, 97, 98].

##### Play friendly supervision

Some participants described positive supervision strategies that accommodated active play and risk taking, such as playground supervisors stepping back, remaining watchful, but intervening in play less [89, 93–99]. These practices aimed to encourage children to think for themselves and make their own judgements, like how high or how fast they could reasonably go in play. As teachers in a Swedish study described “*we want to be the opposite of rules; we want to allow children to test and develop themselves*” ([94], p.6). While a New Zealand principal explained: *“we were saying to them, ‘Oh you can’t play [a game] on those ramps over there.’ And then we suddenly thought, well, you know, step back and watch. Is anyone falling over? Is anyone getting hurt?... And so, we all sort of all said to the teachers, ‘Nah, just let them do it.’ And no one’s been hurt*.” ([93], p.250). However, it was observed in studies that taking a trusting and autonomy supportive approach to risk-taking in play was dependent upon wider factors such as supervision ratios, the state of the physical environment, professional training in relation to play, safety, and risk, and/or sustained support from education authorities and parents [86, 89, 93–98].

#### Physical environment

##### Hard playground surfaces

School staff described several physical environment features that heightened safety concerns and contributed to injuries during recess. Predominantly, these involved hard playground surfaces like concrete, asphalt or compacted soil, and not enough grassed areas or fall-attenuating surfaces around equipment [77, 79, 81, 85, 90, 91, 94]. As described in an English study, teachers’: “*most tangible and pressing… [concerns] were the hard surfaces that tore and bruised flesh and occasionally caused more serious injuries to skulls and bones; the tarmac playground and the brick walls*” ([81], p.254). Additionally, some participants reported that injuries or injury risk increased when new or additional equipment was provided [79, 87]. Interestingly, a Swedish study that explored injury risk in the playground found that despite children being able to engage in risky play activities such as tree climbing and rough and tumble snow fights and war games, the most frequent injury mechanisms observed and/or described by teachers were falls on the same level or collisions [94]. Teachers perceived maintenance issues such as potholes, loose gravel, leaves on the ground, as well as asphalt, were the main contributing factors.

#### Institutions and policy

##### Physical safety focus in policies and rules

An emphasis on children’s physical safety in school policies and rules was described and observed across studies and jurisdictions, which had a constraining effect on children’s active play [75, 79, 82–85, 87, 94, 96, 99]. Safety concerns were reflected in rules that restricted children’s access to play equipment or playground areas [75, 83, 85, 87], and banned games, activities [75, 83, 84, 87, 99], or equipment [75, 94]. Playground injuries could lead to the ‘cause’ of the injury being removed [83, 94]. As a Swedish teacher explained: “*If a child falls from a tree and gets hurt, the tree must probably be cut down*” ([94], p.7). In other cases, a perceived risk of injury was sufficient to warrant restrictive rules or banned activities [82, 85, 87, 94, 101]. As school staff in an English study explained, “*children given free choice often decide on inappropriate games*”, therefore “*children need to follow the rules and understand what they can and can’t do*” ([82], p.14). American yard supervisors described “*student safety and rule enforcement as their biggest priorities*” ([78], p.109), while an English head teacher explained she was “*obliged to protect children from injuring themselves*” and therefore any game at this school considered a contact game was banned ([83], p.56). An Australian teacher summed up the sentiment: “*We’re here for the safety of the children… and that’s paramount in our eyes. And it’s paramount in society’s eyes. So, we have to be careful with the children that we’re entrusted with*” ([96], p.41). Some participants believed this reflected a generational change whereby children in contemporary society faced many more play restrictions and constraints on their learning experiences than their parents and grandparents [94, 96, 97]. Conversely, however, a study with American school nurses found that some perceived principals did not take safety and injury prevention in the playground seriously enough [79].

##### Human and financial resource constraints

Limited human resources, specifically high supervision ratios (e.g., 50:1), were identified as contributing to supervision practices and playground rules that constrained children’s active play [76, 81], such as restricting children’s access to play areas [75, 85] and play equipment [75, 87]. Moreover, high supervision ratios meant staff were not always available to intervene in playground incidents involving bullying or arguments [77, 87] or prevent playground injuries [79, 82]. Authors in a Canadian study observed that insufficient staffing during recess meant yard supervisors and teachers were forced to operate in “*repair mode*” ([87], p.15]. School staff also described how high supervision ratios created a stressful working environment [81, 87]. Additionally, participants explained that limited financial resources reduced schools’ options to address these issues [75, 87] or make physical changes in the playground [87, 88]. For example, Canadian principals reported that staggering recess to address social and behavioural issues and enable better access to play space and equipment was an option they had considered but dismissed due to insufficient funds to hire more supervision staff [87]. Similarly, human and financial resources were perceived to influence the uptake and sustainability of playground interventions that introduced risk and natural elements [88, 93].

##### Institutional risk aversion

In line with participant perceptions of duty of care responsibilities, accountability for children’s safety at the institutional level was also perceived to constrain children’s opportunities for active play in schools. Risk-averse policies and practices were reported both within schools [75, 95, 96], and at the regulatory level e.g., by education authorities [75, 83] and school boards [86, 88]. Participants cited playground safety standards [86, 88], insurance companies [86], and health and safety authorities [83] as drivers for the risk-averse approach in schools. As a Canadian coordinator from a playground naturalisation study described: “*Because of liability issues, the district said you can’t have that, you need to go…you [have to] put that though a landscape architect*” ([88], p.307). While a Canadian teacher from another playground naturalisation study explained: “*There is less enthusiasm from safety officials as they are concerned with lawsuits… but their lack of enthusiasm is often discouraging and can make people fearful of change*” ([86], p.118). Despite legal and regulatory differences across jurisdictions, similar perceptions about institutional risk aversion were described by participants in Australia [96], England [83], Canada [86, 88], and the United States [75]. Furthermore, a cross-cultural comparative study from Sweden and France, observed that differences in the legal framework between countries influenced institutional responses to risk and safety in school play. In France, the school or local education authority was financially liable if a child was hurt in play, and as a result, surveillance and safety of students was the paramount concern during recess, while in Sweden, the municipality provided collective accident insurance for all schoolchildren, and surveillance and safety were a lesser concern among school staff [95].

##### Policy barriers and red tape

Alongside institutional risk aversion, participants described structural barriers such as ‘red tape’ and lack of supportive policy for children’s active play in schools, particularly in relation to implementing or sustaining change [75, 86, 88, 96, 99]. For example, an American principal explained that although children had expressed a desire to use a grassed field adjacent to their school during recess, school policy prevented this because “*using the field was akin to going on a field trip*” for which they did not have the resources to complete required documentation or provide sufficient supervision ([75], p.134). In addition, safety-oriented policies could have the inadvertent effect of protecting one health outcome at the expense of another. For example, sun-smart policies in Australia (e.g., no hat, no play), could negatively affect a child’s active play opportunities by requiring them to sit in the shade during recess [99]. Likewise, wet or cold weather policies often had the effect of constraining active play opportunities by requiring children to stay indoors without the facilities or space to play actively [75, 77, 82, 85, 86]. Such policies were not universal, however, with Icelandic teachers describing all weather play outdoors as a regular feature of school life [92].

##### Teacher/ supervisor education and training

Enhancing the education and training of teachers and playground supervisors was discussed across studies as a path to promoting play and the wellbeing of both children and staff during recess. For example, two Australian studies that evaluated risk-reframing workshops found teachers developed an appreciation of the benefits of risky play which improved their tolerance of risk in the playground [97, 98]. While American teachers participating in a Playworks program (which included staff training and student coaching in pro-social skill development) reported playground conflict decreased and students’ classroom behaviour improved [80]. Moreover, teachers believed the program fostered an increased sense of emotional and physical safety on the playground: As one teacher said, “*there’s a lot more collegiality between the kids. They’re using, ‘hey, good job, nice try,’ instead of ‘ha-ha, you’re out*”’ ([80], p.56). Across studies a range of staff education and training needs were identified, including risk perception [94, 96], injury prevention [79, 94], conflict resolution skills [77, 78], and knowledge/ skills to facilitate active and/or risky play [80, 86, 89]. In some schools, a lack of or inadequate training and guidelines for playground supervision (in addition to a lack of policy for play) created a void whereby personal experiences or attitudes shaped supervision practices rather than professional knowledge or pedagogy [78, 82, 89–96]. Participants and researchers also observed that this contributed to inconsistency among supervisors in the application of playground rules [77, 78, 82, 94], and put pressure on staff with respect to execution of their duty of care responsibilities [75, 77–79, 87, 94]. For example, a Swedish study found teachers attitudes to playground injuries varied across and within schools and were often based on personal experience [94]. Although most teachers in this study distinguished between minor scrapes and bruises incurred as a natural part of play and more serious incidents, definitions of ‘serious’ varied among teachers, with some believing an arm fracture was “*not so bad*”, while others considered this a serious injury ([94], p.7).

##### Balancing benefits against risks in policy and rules

Through formative [92] and program [86, 88, 93, 97, 98] evaluations for school playground interventions, participants described ways their school and staff had worked to balance the benefits and risks in playground policy, overcome barriers to change and improve children’s active play affordances. For example, schools questioned the purpose of playground rules and removed or relaxed rules that weren’t essential [92, 93]. Likewise, schools renegotiated rules with children, seeking a ‘sweet spot’ between enabling children to take risks and challenge themselves and managing the safety concerns of adults [86, 92, 93]. As authors of a New Zealand study described: “*Schools began to reflect upon why they were enforcing certain systems and realised that often there was a default position of ‘no’ rather than simply allowing children to play*” ([93], p.245). Schools also introduced recess policies to promote all-weather (or most weather) play outdoors [86, 88, 93]. Strategies schools employed to balance benefits and risk in policy included involving children in discussions and decision making [88, 92, 93], staff training and play workshops (e.g., risk-reframing) [86, 97, 98], building project committees that included parents, teachers and play ‘champions’ [86, 93], and communicating the benefits to parents [86, 93]. A Canadian school principal explained how her school raised awareness of policy changes by telling parents: “*We really value the time that children spend outside… So, we’re going to be sending your child out if it’s raining lightly… if it’s cold… we’re going out*” ([86], p.114).

#### Society

##### Fear and blame

Across studies, a generalised fear of negative evaluation and blame was described, particularly from parents, but also education authorities, which coincided with a heightened perception of risk for what might happen to children in the playground [81, 83, 86, 88, 89, 96–98]. Some school staff described the emergence of a culture of fear and blame in western society that influenced parental behaviour, supervision practices, and decision making in schools more broadly [83, 86, 96, 98]. As a Canadian teacher discussing barriers to children’s rough and tumble play in school explained: “*What if one of the kids get hurt, and the parents come and say “well, why are you allowing that at the school?””* ([89], p.61). For some school staff, fear of blame or negative repercussions superseded other priorities, including the developmental and wellbeing benefits of active play [86, 94, 96]. For example, an Australian teacher recounted an incident involving a parent who had *“…tried to blame her for an insect flying into the classroom and lodging in a child’s eye”* ([96], p.41). This teacher reported that fear of litigation led her to be more restrictive of children’s play activities during recess than she was of her own children at home. While a Canadian participant voiced: *“I think that we actually really need to start looking at…and tackling this issue of parents and liability and the amount of fear and resistance that it creates within the school setting. We’re placing more value on fear of the parents than on what we inherently know is good for children*” ([86], p.118).

## Discussion

This is the first systematic review of qualitative research to examine adult perspectives on safety and risk in children’s active play during recess in schools. Consequently, it provides needed insight into decision making and supervision practices in schools, and offers direction for parents, teachers, education authorities, and policy makers to promote an active play friendly recess environment. Using the framework synthesis method, a conceptual framework, structured on the SEM and affordance theory, was developed that identified 10 constraining and four affording factors that influenced whether schools and playground supervisors were risk-averse or risk tolerant during recess, and, in turn, the degree to which children’s play was managed. A key finding is that socio-cultural influences in schools have a central role in shaping children’s affordances for active play. Constraining factors stemmed from safety concerns and perceptions of accountability, blame and liability risk in the event of playground injury. These findings were apparent both inside and outside an intervention evaluation context, suggesting more attention should be paid to these wider influences in future efforts to promote children’s active and/or risky play in schools. The synthesis findings are discussed below, together with models that depict potential relationships between factors across SEM levels. As such, this review presents novel insights for school policy managers, educators, and other school personnel striving to promote a positive recess environment that promotes child development and wellbeing through play [102, 103].

### Risk averse decision making and constraining supervision

**Fig. 3:**
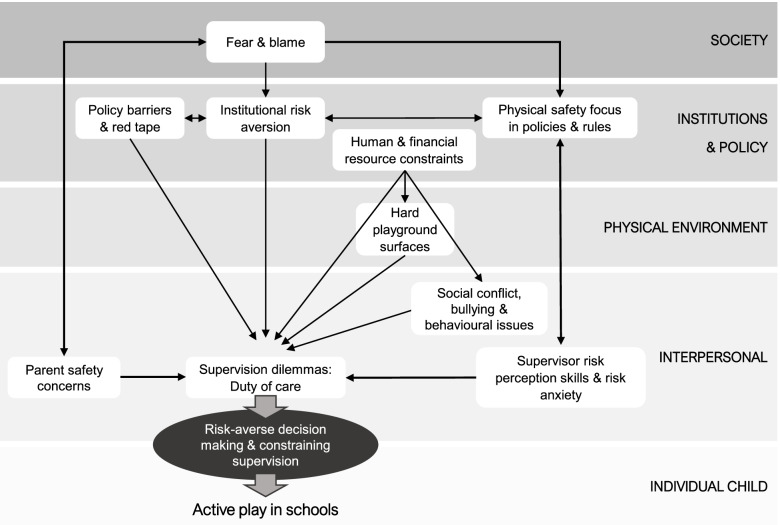
Drivers of risk averse decision making and constraining supervision during recess. Legend: Socio-ecological factors that influence risk-averse decision making and constraining supervision practices during recess in schools

Most factors (10 of 14) limited the ability of schools to provide a recess environment that genuinely promoted active play. Although not readily defined [8], children’s play is any behaviour, activity or process that is freely chosen, self-directed and intrinsically motivated [104], with key characteristics being “fun, uncertainty, challenge, flexibility, and non-productivity” ([9], p.6). The active play definition adopted in this review integrates the characteristics of freely chosen, fun and unstructured ([13], p.164). As depicted in Fig. 3, it was evident in the synthesis that schools and playground supervisors faced a range of interrelated barriers to promoting play of this kind, particularly at the interpersonal, institutional and policy levels. Findings suggest there was a pattern of downward influence through the SEM that culminated around supervision dilemmas in the context of ‘duty of care’ at the interpersonal level and led to risk-averse decision making and constraining supervision in schools.

#### Accountability and legal liability

The drivers for risk-averse decision-making and constraining supervision in schools were perceived to centre around accountability, blame and potential liability in the face of playground injuries. Schools, like other children’s settings, are highly regulated, and participants described layers of bureaucracy and ‘red tape’ regarding school policies and playground design. This contrasts starkly with the lack of policies in schools to promote PA and active play [105], but aligns with the findings of a 2018 white paper examining risk, liability and children’s play in public space [28]. The extent to which either individuals or institutions in the current review faced legitimate litigation risk in relation to children’s injury during play is unclear as the legislative context varies from country to country. For example, in Australia and the United Kingdom (UK), relevant legislation is grounded in the notion of ‘reasonableness’; with the primary task being to reduce the risks ‘so far as is reasonably practicable’ [28]. While in Canada and the US, issues of liability and negligence are more complicated, with leading health organisations calling for legal reform [106]. Nevertheless, research has found liability claims are comparatively uncommon (even in countries with a higher levels of liability claims overall) and there are few examples of case law resulting from playground injuries [28, 107]. Since 2012, the UK Health and Safety Executive (HSE) has attempted to address misunderstandings that persist about liability for play injuries in children’s settings [108]. However, a 2019 UK national study found two of the three main challenges of recess reported by children, were an absence of things to do, and banning of fun activities, with children’s concerns about the latter having *increased* over the previous decade [109]. The degree to which perceptions of liability for playground injuries account for these findings isn’t clear but does require greater understanding, particularly whether perceptions of litigation risk are increasing. A key contributing factor may be the bureaucratic emphasis on paper trails and compliance requirements in western schools that can create an organisational culture where people become more focussed on protecting themselves from negative consequences (of liability or blame) than meeting the developmental and wellbeing needs of children in their care [28]. Moreover, there is emerging evidence that institutional risk aversion in the context of children’s play is also growing in previously more risk tolerant nations such as Norway [110].

#### Parent safety concerns, risk, and blame

Findings indicate parent (or caregiver) safety concerns were another key influencing factor in play provision and supervision practices in schools, yet the voice of parents with respect to risk and safety in play was limited in studies. Across other child domains, parent safety concerns are a well-documented barrier to PA in early childhood (0-6 years) settings and at home [111], older children’s (5-14 years) independent active free play [112], and children’s outdoor play generally [113, 114]. However, there is less evidence regarding parent and caregiver perspectives on active play in schools. A 2019 national survey of New Zealand parents of children aged 5-12 years, found that parents perceived there were too many health and safety rules applied to children’s play in schools [115]. This contrasts with a 2021 review which found school staff concern regarding parent reactions, especially if a child were to be injured playing adventurously, was a barrier to adventurous play [57]. Likewise, research in early childhood settings across western countries, has documented the influence parent safety concerns have on the active play opportunities provided to children [110, 116, 117], and the attitudes to risk-taking in play held by early childhood staff [118]. While the outcomes of the 2021 review [57] centred on school staff perceptions of children and their concerns regarding the safety of adventurous play, the current review has synthesised a wider range of studies using the SEM, seeking to understand the societal, policy, institutional, and interpersonal factors that shape staff perceptions and behaviour. At a societal level, fear of what might happen to children in the playground appeared to drive an emphasis on children’s physical safety that was reflected in supervisor perceptions of risk, parent attitudes and behaviour, and decision-making at the policy and institutional level. According to social anthropologist, Douglas, risk and danger are culturally conditioned ideas, shaped by pressures of social life and accepted notions of accountability [119]. Douglas’ cultural theory of risk proposed that some risks are politicised and elevated in society while others are not, and those that receive more attention relate to legitimating moral principles, and thus can be responded to with fear and anxiety [74]. Such constructions of risk in contemporary western societies are problematic for children’s active play in schools when considering the core features of play (e.g., self-directed, intrinsically motivated, and freely chosen), against the fear risk generates, and the corresponding priority institutions place on surveillance and safety. As such, better understanding of the cultural norms and societal pressures that shape parent safety concerns, their attitudes towards playground injuries in schools, and how parent and institutional responses to risk might be better negotiated, is required.

#### Resource constraints, social conflict, and injuries

**Fig. 4:**
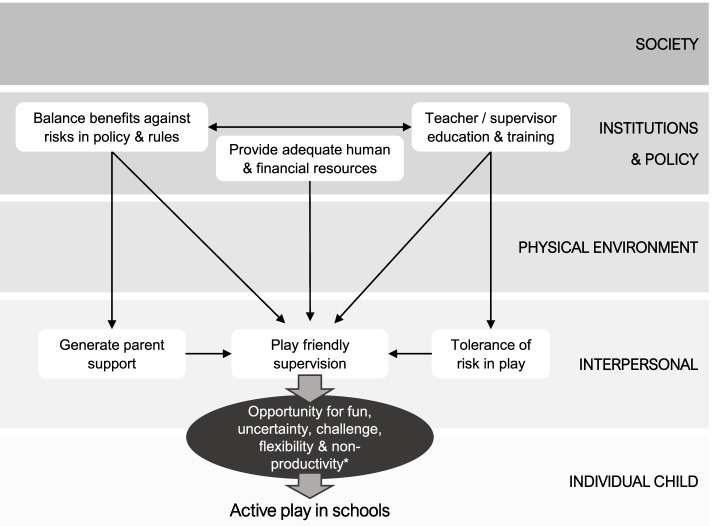
Fostering a risk tolerant and play friendly culture in schools. Legend: Socio-ecological factors that facilitate play friendly supervision and tolerance of risk in active play during recess in schools. *Children’s play is any behaviour, activity or process that is freely chosen, self-directed and intrinsically motivated [104], with the key characteristics being fun, uncertainty, challenge, flexibility & non-productivity [9]

Resource constraints were perceived to contribute to both playground injuries (through hard or poorly maintained surfaces) and social conflict or bullying (through lack of things to do and insufficient supervision staff available to address playground issues). Both of which led to supervision practices and playground rules that constrained active play. In a 2019 UK national study, the biggest concern supervision staff expressed about recess were perceptions of poor student behaviour, and a growing sense this was caused by poor social competence [109]. This is consistent with a 2019 meta-study that examined children’s perspectives on recess that found social factors such as bullying, gender conformity and power hierarchies shaped children’s engagement in play during recess [55]. Institutional responses in some countries to social and behavioural issues during recess have included the introduction of more structured activities for children (e.g., physical education lessons or the ‘daily mile’) and/or less time allocated to recess each day [20, 102, 109, 120]. Such responses, however, are inconsistent with children’s rights to play under Article 31 of the CRC, and may further restrict social development, in addition to wider physical and mental wellbeing effects [20, 121]. To improve understanding, the play affordances available to children in schools where poor social skills and social conflict in the playground are experienced should be examined as a potential contributing factor [75]. Likewise, examination of play affordances and injury incidence is required [93]. Emerging research suggests that introducing more varied, open-ended play environments (that facilitate age-appropriate risk-taking) may reduce social problems (e.g., bullying) and/or playground injuries [96–124]. Mulryan-Kyne contends that although children experiencing inequality or discrimination may benefit from targeted supervision, this should be balanced against the benefits of unstructured, child-led play [121]. An alternative approach, albeit more resource intensive, would be for education authorities to invest in a wider range of play affordances in schools, that are inclusive and meet a diversity of play interests [125] (e.g., asymmetrical playgrounds and natural features that afford more interactions across children of different ages and sizes [126]), coupled with a shift towards a risk tolerant culture (see Fig. 4).

### Fostering a risk tolerant and play friendly culture in schools

The synthesis identified four affording factors that promote children’s active play in schools through development of a risk tolerant culture. These are depicted in Fig. 4 and involve actions at the interpersonal and institutions and policy levels of the SEM. Additionally, parent safety concerns and human and financial resource constraints were identified in the current review as key barriers to an active play friendly recess and are also represented in Fig. 4 (see ‘generate parent support’ and ‘provide adequate human and financial resources’). Teacher/ supervisor education and training to improve tolerance of risk in play and promote play friendly supervision was an important affording factor. Although schools are increasingly regulated settings with respect to child safety, the provision of training for playground supervision is limited and in many countries the pay rates of yard duty supervisors are low, and/or teachers are expected to perform yard duty in addition to their regular duties [78, 82, 102]. This places playground supervisors in the difficult position of being held responsible for the wellbeing and safety of large numbers of children, while not necessarily possessing the requisite skills or receiving adequate pay or support to provide quality supervision [109, 125]. Moreover, in jurisdictions where active play is not part of the curriculum or a valued pedagogical method, teachers and schools may lack incentive to support this aspect of children’s development and wellbeing [9, 103]. One approach may be to map the alignment between active play (and age-appropriate risk-taking) to existing curricula and teaching outcomes (e.g., concentration, problem solving, collaboration, resilience, student wellbeing), to demonstrate the synergies for teachers and build appreciation of the value a play friendly recess environment offers [103]. Additionally, findings indicated the provision of quality playground supervision requires sufficient resources to fund staff education and training, pay suitably qualified staff, and/or improve supervision ratios. These changes, in addition to a more diverse range of play affordances, may allow children more freedom and scope in their play, while also reducing the stress and negative experiences of staff, thereby improving the wellbeing of both [125].

Findings from a small number of playground intervention studies indicated that modifying school policies and rules to balance benefits and risks in play improved children’s active play opportunities and helped generate parent support. Although these studies described informal approaches, a recognised method is Risk Benefit Assessment (RBA), a risk management tool that brings together considerations about both risks and benefits including the benefits that arise as an outcome of the risks [32]. RBA frameworks have been developed in Canada [127], the UK [128], and incorporated into Australian playground safety standards [129]. Indeed, the UK HSE, which oversees safety in schools, recommends a risk-benefit approach to children’s play provision [108]. Another policy-level initiative adopted in Wales, requires Local Authorities to conduct Play Sufficiency Assessments (PSA), which incorporate RBA to evaluate and ensure sufficient and varied outdoor play opportunities for children [130]. PSA requires authorities to examine policies for their role in curtailing children’s play, and training for parents, professionals, and decision makers whose work impacts children’s opportunities to play [130]. To our knowledge, there is little research implementing RBA or PSA approaches in schools, indicating this is an area for future work. Such an approach has been endorsed by the International School Grounds Alliance in their ‘Risk in Play and Learning Declaration’ that includes a call to action for schools to be ‘as safe as necessary, not as safe as possible’ [131]. Findings in the current review indicated that engaging in a process of balancing risks and benefits in policies that influence play also provides schools with an opportunity to positively engage with parents to emphasise the value schools place on outdoor play, explain their policies, and generate parent support.

### Recommendations for policy, practice, and future research

The findings of this review provide important recommendations for policy and practice as well as potential areas for future enquiry.

1. **Foster a risk tolerant and play friendly culture in schools** through:
  Addressing safety concerns in schools through staff (and parent) education and training, on topics identified by stakeholders at the local level, such as risk perception, risk-reframing, injury prevention, or conflict resolution skills.
  Incorporating theory and practice modules in teacher pre-service education for facilitating children’s active play and understanding the synergies between active play, age-appropriate risk-taking, and learning outcomes in schools.
  Engaging parents, children, school staff and policy-makers, in the development and dissemination of policy that balances benefits against potential risks in active play, using methods such as risk benefit assessment.
  Working with policy-makers at the education authority and/or school level to reduce red tape and overcome policy barriers and liability fears to provide a recess environment that supports children’s age-appropriate risk-taking in play.
2. **Build support to address resource shortcomings for recess in schools** through collaborative research and advocacy that makes the case for affordance-rich play environments in schools [125, 132]. A rights-based approach to play, based on the recommendations of the UN-CRC, should underpin this work [9]. A 2020 Position Statement on Recess in Canadian Elementary Schools provides a working example [102].
3. **Investigate parent attitudes to risky play and children’s safety in schools** and explore how parent and institutional responses to risk might be better understood and negotiated to foster tolerance of risk in active play outdoors.
4. **Examine the influence of diverse and open-ended play affordances in schools** (that facilitate risk-taking and challenge) on social conflict and injury incidence in the playground.

### Strengths and limitations

Major strengths of this review include rigorous application of the framework synthesis method, and development of conceptual framework grounded in theory and empirical evidence. Reflecting the multi-disciplinary nature of the topic, multiple databases were systemically searched to generate a breadth of perspectives. Additionally, incorporating the perspectives of all adults with a ‘stake’ in children’s active play in schools provided a systems-level analysis of the contemporary issues schools face, from the perspectives of those on the ground [133]. The search strategy did not, however, include studies published in languages other than English or grey literature (an acknowledged deviation from the registered protocol based on the number of studies and volume of data identified in the peer-reviewed literature) and possibly important information was overlooked. Moreover, it may be that limitations exist in the evidence, and there are further influencing factors in school systems not yet understood. Indeed, the perspectives of parents were under-represented in studies, only two studies were conducted in middle schools, and studies did not always provide sufficient contextual or demographic information for participants such as school characteristics, participant gender, qualifications, or experience. Moreover, 80% of studies were conducted in anglosphere nations, therefore transferability of findings to other countries and cultures, should be made cautiously. Additionally, researcher positionality and critical examination of the potential for influence or bias during the research process was not consistently examined in the primary studies. Although reflexivity was used in the current review, the authors acknowledge the role and potential influence our respective backgrounds may have had in the review process, in particular the underlying value shared by authors that active play in schools is important to foster, even if this entails risk.

## Conclusions

This systematic review synthesised qualitative research that examined how parents, teachers, yard supervisors, and principals view safety and risk in children’s active play during recess in schools. Using the framework synthesis method, a conceptual framework structured on the SEM and affordance theory was developed, comprising 10 constraining and four affording factors. Findings show socio-cultural factors in schools have a central role, with several factors restricting the ability of schools to genuinely promote active play. Constraining factors stemmed from fears for children’s physical safety, and fear of blame and liability in the event of playground injury, which shaped parent, school staff and institutional responses to risk. Interrelated factors across SEM levels combined to drive risk-averse decision making and constraining supervision during recess. Emerging research suggests that children’s active play can be promoted by fostering a risk tolerant and play friendly culture in schools through teacher/supervisor education and training and engaging all stakeholders (including children) in the development of school policies and rules that balance the benefits of play against potential risks. Such changes may help address parent, supervisor, and institution-level concerns regarding safety and negative playground behaviour. Future work should seek to understand and challenge the cultural norms that shape parent attitudes and institutional responses to risk in children’s play and explore novel methods for overcoming policy barriers and liability fears in schools.

## Supplementary Information

**Additional file 1.** PRISMA Statement & ENTREQ Checklist. Description: The completed statement and checklist.

**Additional file 2.** Search strategy. Description: The search strategy, key concepts and search terms, and example database search.

**Additional file 3.** Initial conceptual framework and codebook. Description: An explanation of the development process for the initial conceptual framework, together with the codebook that guided the evidence synthesis.

**Additional file 4.** Characteristics of included studies. Description: Study characteristics table, including Author, year, country, discipline, research aim, study design, theoretical framework, sampling methods, setting and participant characteristics, data collection and analysis methods, rigour.

**Additional file 5.** Quality appraisal of included studies. Description: Appraisal results table for all studies using the CASP Qualitive Checklist.

## Data Availability

All data generated or analysed during this study are included in this published article and its supplementary information files.

## Abbreviations

ENTREQ: Enhancing Transparency in Reporting the Synthesis of Qualitative Research statement
CASP: Critical Appraisal Skills Programme Qualitative Checklist
CRC: United Nations Convention on the Rights of the Child
HSE: Health and Safety Executive
PA: Physical activity
PSA: Play Sufficiency Assessments
PRISMA: Preferred Reporting Items for Systematic Reviews and Meta-Analyses checklist
RBA: Risk Benefit Assessment
SEM: Socio-ecological model
UK: United Kingdom
UN-CRC: United Nations Committee of the Rights of the Child
US: United States
