# Supplementary material for: “Children are precious cargo; we don’t let them take any risks!”: Hearing from adults on safety and risk in children’s active play in schools: a systematic review
Source: Int J Behav Nutr Phys Act. 2022 Sep 1;19:111. doi: 10.1186/s12966-022-01344-7 (PMC9438168; doi:10.1186/s12966-022-01344-7)
Supplement: Supplementary file 3 — Additional file 3. Initial conceptual framework and codebook. Description: An explanation of the development process for the initial conceptual framework, together with the codebook that guided the evidence synthesis. [file 12966_2022_1344_MOESM3_ESM.docx]

# Additional file 3: Initial conceptual framework & codebook

Note: The process undertaken to develop the initial conceptual framework and codebook is applicable to two systematic reviews, concerning a) adults’ perspectives (the current review), and b) children’s perspectives (a separate review) on the role of safety and risk in children’s active play during recess in schools. The codebook (and themes derived for indexing) were adapted for the current review.

## Adapted codebook for review of adult perspectives: parents, teachers, playground supervisors, administrators, and other school staff

| **Social Ecological Model (SEM)** | **Domain** | **Themes derived for Indexing** | **Definition** | **Coding Notes / Application to this review** |
| --- | --- | --- | --- | --- |
| **Individual level** | **Demography** | 1. Age & gender | How age and gender influence children’s active play behaviour, engagement in risk and safety management | Observed preferences between Girls vs. boys, influence of age in how children interact with affordances and each other. |
|  | **Physical literacy** | 1. Physical Literacy | - Physical, psychological, social or cognitive factors that shape safety and risk behaviours in play - PL outcomes of risky play | Categorise according to Australian PL Framework  Relationship between physical skills and fear of injury or risk-taking and play preferences. Relationship between confidence and risk-taking in play. Cooperation and Negotiation skills that help children solve potential injury or conflict situations. The reciprocal role between play and the development of risk perception and risk management skills. |

| **SEM** | **Domain** | **Themes** | **Definition** | **Coding Notes / Application to this review** |
| --- | --- | --- | --- | --- |
| **Interpersonal level** | **Family** | 1. Parents’ safety concerns | The influence of parents’ safety concerns on:   - children’s play affordances in schools - supervisor perceptions of active play and supervision practices | Parents reported concerns and/or strategies to address parent concerns  Teacher/ administrator perceptions of parents’ safety concerns and/or strategies to address parent concerns |
|  | **Peers** | 1. Social conflict and bullying | The influence of social conflict and bullying on:   - children’s play affordances in schools - supervisor concerns and supervision practices | Includes both the impact of bullying (making children feel unsafe) and the potential causes of conflict and bullying (lack of affordances, too many constraints).  Children’s conflict resolution skills |
|  |  | 1. Friendship and belonging | The influence of friendship and belonging on children’s play affordances in schools. | Children having someone to play with opens up affordances, feeling socially safe, having friends to stick up for you. |
|  | **Supervisors** | 1. Constraining supervision practices | Supervision practices that constrain children’s active play at school. | May not need this one for adult review? Potential cross over with ‘supervision dilemma’s’? |
|  |  | 1. Play friendly supervision | Supervision practices that afford children’s active play at school. | Supervision practices that support risk-taking and challenge in active play |
|  |  | 1. Supervision dilemmas | Supervision dilemmas reported by teachers and playground supervisors leading to constraining behaviours. | There are a number explicitly reported in the literature (fear of litigation), and latently in adults’ attitudes to safety (fear of negative evaluation, blame). |
|  |  | 1. Supervisor risk perception | The influence of supervisors’ risk perception on children’s active play affordances. | Both constraining factors (risk anxiety, low risk tolerance) & affording factors (teacher strategies for managing risk in play, teachers’ recognition of children’s risk management abilities).  Question of teachers own physical literacy in how they assess safe or unsafe play? |
|  |  | 1. Supervisor attitudes and beliefs | The influence of supervisors’ attitudes and beliefs on constraining or supporting active play. | Beliefs about play. Constructs of children – fundamentally resilient or fundamentally vulnerable. |

| **SEM** | **Domain** | **Themes** | **Definition** | **Coding Notes / Application to this review** |
| --- | --- | --- | --- | --- |
| **Physical environment level** | **Physical affordances** | 1. Spatial constraints | The influence of playground space and density on children’s play affordances from the perspective of risk and safety. | Safety concerns relating to playground space and density. Children’s use of non-play features in play to add challenge and interest. |
|  |  | 1. Playground surface safety | The influence of playground surfaces on children’s play affordances from the perspective of risk and safety. | Safety concerns relating to playground surfaces (asphalt vs grass, adequate fall surfaces). |
|  |  | 1. Equipment, fixed and loose | The influence of loose and fixed equipment on children’s play affordances from the perspective of risk and safety. | Safety concerns relating to poor maintenance.  Lack of age-appropriate challenge in equipment |
|  |  | 1. Natural environment | The influence of natural features on children’s play affordances from the perspective of risk and safety. | Open-ended play opportunities of natural features increase affordances, but also less safe. |
| **SEM** | **Domain** | **Themes** | **Definition** | **Coding Notes / Application to this review** |
| **Policy and institutional level** | **School culture** | 1. Types and Reasons for restrictive rules | The reasons offered for restrictive rules. | Is this *physical safety focus in policies and rules*?  what about the drivers for concern with physical safety? |
|  | **Education system** | 1. Regulation and legislation | The external policy-level influences on the school play environment and children’s play affordances. | Systemic-level issues relating to safety and risk management that constrain play such as legally structured policies (duty of care), playground design guidelines.  Lack of legislation and policy to protect play. |
|  |  | 1. Resources | The financial resources available to schools for playground design, number and qualifications of supervision staff. | Low supervision ratios drive safety focus.  Under resourced playgrounds. |
|  |  | 1. Teacher and supervisor education | Staff training and education influences on the play environment and children’s play affordances. | Methods for supporting play through risk reframing; risk benefit analysis (RBA); dynamic RBA. Injury prevention training. |
| **SEM** | **Domain** | **Themes** | **Definition** | **Coding Notes / Application to this review** |
| **Societal level** | **Social and cultural norms** | 1. Cultural nature of acceptable risk in play | The cultural embeddedness of risk in play for example, differences between nations in attitudes to risk in play and what is or is not ‘safe’ | Evidence for differences between countries in attitudes to risk in play and what is acceptable in schools. |
|  |  | 1. Societal aversion to risk | The influence of societal risk aversion on children’s play affordances. | Evidence for dominance of safety culture (e.g. surplus safety), fear of blame or negative consequences in society that strongly influences decision making in schools. |

## Initial conceptual framework

Each of the 25 initial themes can be conceptualised in terms of ‘affording’ or ‘constraining’ children’s active play.

| **Social Ecological Model** | **Individual** | **Interpersonal** | **Physical environment** | **Policy and institutional level** | **Societal level** |
| --- | --- | --- | --- | --- | --- |
| **Description** | Risk and safety influences on active play at the Individual Child level | Risk and safety influences at the Interpersonal level | Risk and safety factors in the physical environment at school | Risk and safety factors at the policy and institutional level | Social and cultural norms around risk and safety in play |
| **10 DOMAINS**  **&**  **25 Themes** | RISKY PLAY INCLINATION   1. The play appeal of risk-taking and challenge 2. Types of risky play children wish for   PHYSICAL LITERACY   1. Physical, Psychological, Social and Cognitive skills, capacities and attributes   DEMOGRAPHY   1. Demographic factors | FAMILY   1. Parents’ safety concerns   PEERS   1. Bullying in the playground 2. Social conflict in the playground   SUPERVISORS   1. Playground supervisor characteristics 2. Supervision practices 3. Supervision dilemmas 4. Supervisor pedagogical Knowledge 5. Supervisor risk perception and management skills 6. Supervisor attitudes and beliefs | PHYSICAL AFFORDANCES   1. Playground space and density 2. Playground surfaces 3. Equipment, fixed and loose 4. Natural features | SCHOOL CULTURE   1. School policies and rules 2. Child perspectives in policy and playground design   EDUCATION SYSTEM   1. Regulation and legislation 2. Resources 3. Education pedagogy and play 4. Teacher and supervisor education | SOCIAL & CULTURAL NORMS   1. Cultural nature of risk 2. Societal aversion to risk |

## Development of the initial conceptual framework and codebook

A framework synthesis begins with an initial conceptual framework to guide the extraction and combination of the data [1]. The initial conceptual framework was systematically developed through the following steps:

1. Familiarisation and becoming immersed in the data
2. Identification of relevant literature
3. Identification of relevant theory
4. Compiling definitions
5. Mapping and categorising themes and concepts into a thematic map
6. Synthesising and re-organising thematic map into pilot codebook and initial conceptual framework
7. **Familiarisation and becoming immersed in the data**
   1. Several activities contributed to this step, including 1^st^ and 2^nd^ stage full-text screening. Additionally, prior to the 2^nd^ stage screening, study characteristics for the 70 studies included at stage 1 were extracted. These studies were then re-read and evaluated against the additional eligibility criterion described in the Methods section to determine whether they provided ‘contextually thick’ findings in relation to the review question.
8. **Identification of relevant literature**
   1. The abstracts of studies included at the end of stage 2 full-text screening and selection (n=41) were examined to identify which studies had explicitly investigated risk or safety in children’s play at school. This identified nine studies [2-10], and the themes and findings of each study were extracted.
   2. The reference lists of included studies were examined for qualitative reviews of factors that shape children’s active play and/ or risky play in schools. This identified three reviews [11-13], and one additional review that examined the determinants of children’s independent active free play [14]. The results of these reviews were extracted, and findings or themes relating to risk or safety were extracted.
   3. An additional review [15], examining perceived barriers and facilitators to adventurous play in schools, published during framework development was judged relevant to the Review Question and the key findings were subsequently included and extracted.
   4. A search was conducted in Scopus for highly influential papers (as measured by citations) using a) the key words “risky play” and b) the search string “children AND safety AND active play”. This identified five additional articles relevant to the review question [16-20], and findings and themes of these studies were extracted.
9. **Identification of relevant theory**

From the 18 articles above, relevant theory to guide the framework development was identified:

- 1. Social-ecological model (SEM)

The SEM was chosen as an organising structure in the framework. The SEM was the most frequently employed theoretical framework in the articles, including all four reviews. Developed from Bronfenbrenner’s [21] ecological systems theory, the key principle of the SEM is that there are multiple levels of influence on health behaviours, including intrapersonal factors, (psychological, biological), interpersonal (cultural, social), physical environment, organisational, policy and societal factors [22]. The SEM is particularly well-suited for studying children’s active play because it incorporates people’s interactions with their social and physical environment, while also recognising the influence of policy and societal factors [23]. Moreover, the influences on behaviour interact across these different levels, meaning that multi-level interventions to address the determinants of behaviour change are likely to be the most effective [22]. Therefore, using the SEM to structure the conceptual framework will enable identified safety and risk factors to be examined according to their level of influence and may provide deeper insight into the relationship between children’s active play behaviour and the social, physical, and policy environment in schools.

- 1. Theory of Affordances

Gibson’s [24] theory of affordances was frequently employed in articles that explored children’s risk-taking in play [19, 20], and provides a useful basis for examining how children interact with their environment. Affordances describe the ‘functional possibilities’ that the environment, and objects in the environment, can provide to an individual. Gibson argued that we perceive the environment in terms of the behaviour it affords, that is, what it ‘‘invites’’ us to do [24]. The concept of affordances includes both the environment and the person, meaning that affordances are unique for each individual and correspond with the individual’s body size, strength, skills, courage, fear, etc. Gibson [24] also argued that other individuals offer affordances by inspiring or constraining actions. The theory of affordances has been extended in recent decades in relation to children’s play, most notably by Kytta [25], who distinguished between potential affordances (a feature of the environment that can be perceived by an individual) and actualised affordances (a feature of the environment that an individual perceives, utilises, or shapes). Kytta argued that children’s ability to perceive affordances develops systematically as they grow and learn new physical skills, and their ability to use or shape affordances for play is often regulated by their social environment through fields of promoted (culturally defined and socially approved affordances) and constrained action (affordances constrained by others or inherently through their design) [25]. Therefore, the conceptualisation of risk and safety factors that shape children’s active play in terms of ‘affording’ or ‘constraining’ behaviour provides a useful lens to aid understanding of influences on behaviour in the school setting.

1. **Compiling definitions**

Based on definitions employed in the articles identified for the development of the conceptual framework, the following definitions were adopted:

**Active play*:*** *‘a form of gross motor or total body movement in which children exert energy in a freely chosen, fun, and unstructured manner’* [26]. In recognition of the inconsistency in the literature in relation to defining and measuring active play, in 2017, Truelove and colleagues [26] systematically reviewed the literature to identify key concepts used to define and describe active play among young children (0-6 years), with the aim of proposing a working definition for consideration. Notably this definition is the first to highlight the *freely chosen and fun* elements of active play, which may be important for understanding the influences on children’s play behaviour.

**Risky play:** *‘thrilling and exciting forms of play that involve uncertainty and a risk of physical injury’* [27, p.22]. Sandseter identified 6 categories of risky play: play at heights, play at speed, rough and tumble play, play near dangerous elements, play with tools, play where children can disappear or get lost [19]. These categories have since been expanded upon by scholars working with both younger and older children [28-30]. Table 3 describes 10 categories of play that involve risk and how they might apply in the school setting. Notably, the category of ‘Vicarious play’ (defined as, children observe other children taking risks in play, and where the observing child shows clear signs of being exhilarated by what he or she observes) [28] was omitted, as we determined it did not pertain to ‘active play’ and would be difficult to identify from the literature. However, we identified an additional risky play type, ‘body play’ (defined in Table 3), based on our initial reading of the literature.

**Risk perception:** *‘the subjective assessment of the probability of a specified type of accident happening and how concerned we are with the consequences. To perceive risk includes evaluations of the probability as well as the consequences of a negative outcome’* [31]. Risk perception focuses on the perception and appraisal of a situation before deciding to act and is therefore part of risk competence or risk management [32].

**Risk management skills*:*** *‘where an individual has the competence to see opportunities in a risky situation and consider either: (1) to engage in ground-breaking activities; (2) to transform it in a more manageable/controllable situation; or (3) not to participate.’* [32]. Also referred to as ‘risk competence’.

**Physical literacy**: is defined by the International Physical Literacy Association as, ‘*the motivation, confidence, physical competence, knowledge and understanding to value and take responsibility for engagement in physical activities for life*’ [33]. Although physical literacy can be developed at any age, childhood is identified as an important stage for the accumulation of movement experiences that contribute to physical literacy [34]. To promote a shared understanding and language about physical literacy in Australia, the Government agency, Sport Australia, worked with a diverse range of stakeholders across education, sport, health and academic fields to develop the Australian Physical Literacy Framework. The Australian framework adopts a holistic conception of physical literacy, made up of 30 elements across four domains: physical, psychological, social and cognitive [35]. Physical literacy was included in the conceptual framework for this review to reflect the reciprocal relationship between active play and physical literacy: through active play, children develop physical literacy, and children draw on physical literacy, and the range of skills, capacities, and attributes it encompasses, when taking risks in play and keeping themselves safe.

1. **Mapping and categorising themes and concepts**
   1. The themes and relevant findings across all papers were mapped and categorised according to the SEM, with consideration to a) affording and constraining factors, and b) participant voice e.g. child versus adult perspective. This resulted in 24 categories.
2. **Synthesising and re-organising thematic map into pilot codebook and initial conceptual framework**
   1. The thematic map was converted into a first draft codebook for piloting with a subset of review papers. Changes arising from piloting include:
      1. The child / adult distinction was collapsed into one, with some themes relating to either child or adult perspective, and some themes relating to both child and adult perspective.
      2. The framework became structured with the child at the centre of the SEM – originally both child [11, 12, 14, 19, 20] and adult [6, 8, 9, 13] were at the centre of the SEM, based on reviews and research articles included, which didn’t work for coding.
      3. An additional theme was introduced to distinguish between a) the play appeal of risky play and b) the types of risky play children desire. This took the number of themes to a total of 25.
      4. Themes names and definitions were refined and organised into Domains. Coding notes and examples were included to guide further coding. This was an iterative process that continued throughout data extraction and analysis.
      5. The refined codebook was distilled down to the initial conceptual framework.

# References

1. Gough, D., S. Oliver, and J. Thomas, *An Introduction to Systematic Reviews. 2nd Edition*. 2nd edition. ed. 2017, London: SAGE Publications Ltd.

2. Bundy, A.C., et al., *The risk is that there is ‘no risk’: a simple, innovative intervention to increase children’s activity levels.* International Journal of Early Years Education, 2009. **17**(1): p. 33-45.

3. Christensen, P. and M.R. Mikkelsen, *Jumping off and being careful: Children's strategies of risk management in everyday life.* Sociology of Health and Illness, 2008. **30**(1): p. 112-130.

4. Farmer, V.L., et al., *What did schools experience from participating in a randomised controlled study (PLAY) that prioritised risk and challenge in active play for children while at school?* Journal of Adventure Education and Outdoor Learning, 2017. **17**(3): p. 239-257.

5. Gyllencreutz, L., et al., *Injury risks during outdoor play among Swedish schoolchildren: teachers’ perceptions and injury preventive practices.* Education 3-13, 2020. **48**(1): p. 1-11.

6. Hudson, S.D., H.M. Olsen, and D. Thompson, *An Investigation of School Playground Safety Practices as Reported by School Nurses.* Journal of School Nursing, 2008. **24**(3): p. 138-144.

7. Hyndman, B.P. and A. Telford, *Should educators be ‘wrapping school playgrounds in cotton wool’to encourage physical activity? Exploring primary and secondary students’ voices from the school playground.* Australian Journal of Teacher Education, 2015. **40**(6): p. 60-84.

8. Niehues, A.N., et al., *Reframing healthy risk taking: Parents’ dilemmas and strategies to promote children’s well-being.* Journal of Occupational Science, 2016. **23**(4): p. 449-463.

9. Niehues, A.N., et al., *Everyday uncertainties: reframing perceptions of risk in outdoor free play.* Journal of Adventure Education and Outdoor Learning, 2013. **13**(3): p. 223-237.

10. Peterson, S.S., et al., *Children's Rough and Tumble Play: Perspectives of Teachers in Northern Canadian Indigenous Communities.* Early Years: An International Journal of Research and Development, 2018. **38**(1): p. 53-67.

11. Massey, W., L. Neilson, and J. Salas, *A critical examination of school-based recess: what do the children think?* Qualitative Research in Sport, Exercise and Health, 2019.

12. Hyndman, B.P., A. Benson, and A. Telford, *Active play: exploring the influences on children's school playground activities.* American Journal of Play, 2016. **8**(3): p. 325.

13. Van Rooijen, M. and S. Newstead, *Influencing factors on professional attitudes towards risk-taking in children’s play: a narrative review.* Early Child Development and Care, 2017. **187**(5-6): p. 946-957.

14. Lee, H., et al., *A meta-study of qualitative research examining determinants of children's independent active free play.* International Journal of Behavioral Nutrition and Physical Activity, 2015. **12**(1).

15. Nesbit, R.J., et al., *Perceived Barriers and Facilitators of Adventurous Play in Schools: A Qualitative Systematic Review.* Children (Basel, Switzerland), 2021. **8**(8): p. 681.

16. Wyver, S., et al., *Ten ways to restrict children's freedom to play: The problem of surplus safety.* Contemporary Issues in Early Childhood, 2010. **11**(3): p. 263-277.

17. Brussoni, M., et al., *Risky Play and Children's Safety: Balancing Priorities for Optimal Child Development.* International Journal of Environmental Research and Public Health, 2012. **9**(9): p. 3134-3148.

18. Tremblay, M., et al., *Position statement on active outdoor play.* International Journal of Environmental Research and Public Health, 2015. **12**(6): p. 6475-6505.

19. Sandseter, E.B., *Categorising risky play—how can we identify risk‐taking in children's play?* European Early Childhood Education Research Journal, 2007. **15**(2): p. 237-252.

20. Sandseter, E.B.H., *Affordances for risky play in preschool: The importance of features in the play environment.* Early Childhood Education Journal, 2009. **36**(5): p. 439-446.

21. Bronfenbrenner, U., *Ecological models of human development.* Readings on the Development of Children, 1994. **2**(1): p. 37-43.

22. Sallis, J.F., N. Owen, and E.B. Fisher, *Chapter 20: Ecological models of health behaviour*, in *Health behavior and health education: theory, research, and practice*, K. Glanz, et al., Editors. 2008, John Wiley & Sons: San Francisco.

23. Brusseau, T., S.J. Fairclough, and D.E. Lubans, *The Routledge Handbook of Youth Physical Activity*. 2020, New York: Routledge.

24. Gibson, J.J., *The Ecological Approach to Visual Perception : Classic Edition*. 1979, London: Taylor & Francis Group.

25. Kyttä, M., *The extent of children's independent mobility and the number of actualized affordances as criteria for child-friendly environments.* Journal of Environmental Psychology, 2004. **24**(2): p. 179-198.

26. Truelove, S., L.M. Vanderloo, and P. Tucker, *Defining and measuring active play among young children: A systematic review.* Journal of Physical Activity and Health, 2017. **14**(2): p. 155-166.

27. Sandseter, E.B.H., *Scaryfunny: A Qualitative Study of Risky Play Among Preschool Children*, in *Department of Psychology*. 2010, Norwegian University of Science and Technology: Trondheim.

28. Kleppe, R., E. Melhuish, and E.B.H. Sandseter, *Identifying and characterizing risky play in the age one-to-three years.* European Early Childhood Education Research Journal, 2017. **25**(3): p. 370-385.

29. Jelleyman, C., et al., *A cross-sectional description of parental perceptions and practices related to risky play and independent mobility in children: The New Zealand state of play survey.* International Journal of Environmental Research and Public Health, 2019. **16**(2).

30. Brussoni, M., et al., *What is the relationship between risky outdoor play and health in children? A systematic review.* International Journal of Environmental Research Public Health, 2015. **12**(6): p. 6423-6454.

31. Sjöberg, L., B.-E. Moen, and T. Rundmo, *Explaining risk perception. An evaluation of the psychometric paradigm in risk perception research*. 2004, Norwegian University of Science and Technology: Trondheim, Norway.

32. Lavrysen, A., et al., *Risky-play at school. Facilitating risk perception and competence in young children.* European Early Childhood Education Research Journal, 2017. **25**(1): p. 89-105.

33. International Physical Literacy Association. *International Physical Literacy Association homepage*. 2017 14 May 2020]; Available from: <https://www.physical-literacy.org.uk>.

34. Gallahue, D.L., J.C. Ozmun, and J. Goodway, *Understanding motor development : infants, children, adolescents, adults*. Seventh edition. ed. 2012: McGraw-Hill.

35. Sport Australia, *Australian Physical Literacy Framework*. 2019, Australian Sports Commission: Canberra.
