# Supplementary material for: “Children are precious cargo; we don’t let them take any risks!”: Hearing from adults on safety and risk in children’s active play in schools: a systematic review
Source: Int J Behav Nutr Phys Act. 2022 Sep 1;19:111. doi: 10.1186/s12966-022-01344-7 (PMC9438168; doi:10.1186/s12966-022-01344-7)
Supplement: Supplementary file 4 — Additional file 4. Characteristics of included studies. Description: Study characteristics table, including Author, year, country, discipline, research aim, study design, theoretical framework, sampling methods, setting and participant characteristics, data collection and analysis methods, rigour. [file 12966_2022_1344_MOESM4_ESM.docx]

# Additional file 4: Characteristics of included studies

| **Author, Year,**  **Country** | **Discipline** | **Research Aims** | **Study Design & Theoretical Framework** | **Sampling Methods:**  **School Setting & Participants** | **School Setting Characteristics** (n=sample size) | **Participant Characteristics** (n=sample size) | **Qualitative Data Collection** | **Qualitative data analysis** | **Rigour** |
| --- | --- | --- | --- | --- | --- | --- | --- | --- | --- |
| Bundy et al. 2009 [1],  **Australia** | Public health: PA promotion | To examine teachers' perceptions regarding the benefits and consequences of changing the level of risk in the school ground. | Intervention evaluation, Mixed methods | Schools: NR  Participants: Principal nominated teachers that represented opinions held by all 30-school staff. | Elementary (n=**1**), urban, Sydney. | Teachers (n=**9**),  100% female. | Individual and paired Interviews, semi-structured | Constant comparative analysis | Investigator triangulation, collaborative review of themes |
| Farmer et al. 2017 [2],  **New Zealand** | Public health: PA promotion | To examine school staff's perceptions of an intervention that that increased opportunity for risk and challenge in active outdoor play. | Intervention evaluation (acceptability) | Schools: Recruited as part of Play Study RCT.  Participants: NR but included 1-2 leaders from each Intervention school. | Elementary (n=**8**), urban, Auckland and Otago regions,  Low & Med SES. | Principals/ Administrators, (n=**10**)  40% female. | Interviews, semi-structured (Individual or paired),  Field Notes, Reflective research diary | Content analysis, using abductive approach | Reflective research diary, peer debrief |
| Graham et al. 2022 [3],  **England *** | Public health: PA promotion | To determine playground users (primary school staff and pupils) perceptions of the barriers and facilitators to a physically active school playground. | SEM | Schools: Purposive based on low SES and convenience based on proximity of location  Participants: Convenience - all staff with role in PA promotion invited | Elementary (n=**3**), urban,  low SES | Staff with role in PA promotion, including PE specialists, teachers, head teachers, playground supervisors (n=**11**) | Questionnaire with open-ended questions | Thematic analysis, using Braun & Clarke’s 6-step process | Data source triangulation,  Investigator triangulation,  collaborative review of themes |
| Gyllencreutz et al. 2020 [4],  **Sweden ^ⴕ^** | Injury prevention | To explore injury risk situations among school children during  outdoor play in the school environment | Field study design,  Theory of social representations | Schools: Purposive based on playground type.  Participants: Convenience sampling, all children and teachers from relevant grades invited to participate. | Elementary (n=**2**), urban, Sub-artic Northern Sweden,  Small forest, ice skating rinks, tree houses in schoolyard. | Teachers (n=**28**),  2-20+ years’ experience. | FG,  Naturalistic observations,  Field notes | Content analysis, using an observation scheme and play coding structure | Data source triangulation, method triangulation |
| Harper et al. 2019 [5],  **Canada** | Environmental health and education | To explore the process of naturalizing a schoolyard with school stakeholders involved in project planning and implementation. | Instrumental case study approach [evaluation] | Schools: Recruited as part of a school naturalization project.  Participants: Purposive sampling of adult stakeholders involved in project. | Elementary (n=**1**) and Middle (n=**1**), urban, Vancouver Island,  Schoolyards had undergone naturalization. | Principals/ Administrators (n=**2**),  Teachers (n=**4**),  Other staff (n=**4**),  Parent (n=**1**),  73% female. | FG, semi-structured (Middle school),  Interview, semi-structured (Elementary school) | Thematic analysis, using inductive coding approach | Investigator triangulation, collaborative review of themes |
| Hudson et al. 2008 [6],  **USA** | Injury prevention | To investigate school playground safety practices with school nurses. | NR | Schools: N/A  Participants: Purposive sampling of attendees (school nurses) at an educational session about playground safety at the 2006 NASN conference. | Elementary,  across 25 states (n=**NR**), urban,  Participants worked at up to 4 schools each. | School nurses (n=**64**),  Mean 11 years’ experience as school nurse. | Questionnaire with open and closed-ended questions | Data coded inductively; themes generated using constant  comparative method | NR |
| Jarvis et al. 2007 [7],  **England ^ⴕ^** | Child development and play | To investigate the narratives underpinning rough and tumble play in the early years of primary school. | Ethnography,  Bio-culturalism | Schools: NR  Participants: NR | Elementary (n=**1**),  urban,  Northern England. | Teachers (n=5).  Playground Supervisors (n=**NR**). | Interviews, semi-structured, Observations,  Field notes,  Informal conversations | NR | 18-month immersion in the research context |
| Kuru et al. 2020 [8],  **Turkey** | Child development and play | To reveal the opinions of primary school teachers and school managers about the evaluation of primary school gardens for children. | Phenomenology | Schools: Purposive, maximum diversity sampling.  Participants: Purposive, maximum diversity sampling (adults),  random sampling (children). | Elementary (n=**6**),  Muş province. | Teachers (n=**30**),  67% female.  Principals/ Administrators (n=**12**), 25% female. | Interviews, semi-structured | Content analysis, using inductive coding | Data source triangulation, Investigator triangulation, inter-rater reliability |
| Larsson et al. 2021 [9],  **Sweden & France *** | Child development and play | To explore teachers’ and principals’ opinions and everyday uses of the schoolyard, including similarities and differences between countries. | Cross-cultural explorative & comparative design.  Socio-cultural framework  Lefebvre’s social production of  space. | Schools: Purposive in Sweden via direct invitation; Convenience in France via municipal gatekeeper.  Participants: NR | Elementary (n=**10**), urban and rural. | Teachers (**n=4**; 2 per country) majority female.  Principals/ Administrators (**n=6**; 3 per country) majority female. | Semi-structured walking interview (school yard),  Playground observation | Thematic analysis named but method not described | NR |
| London et al. 2015 [10]  **USA *** | Child development and play | To examine how implementing a high-functioning recess can contribute to a positive overall school climate in low-income elementary schools. | NR | Schools: Purposive based on low income and implementation of Playworks program  Participants: NR | Elementary, urban, inner city (n=6).  73% students Latino, 60% English learner, 78% free lunches. | Principals/ Administrators (n=**6**)  Teachers (n=**15**)  Recess coach (n=**6**) | Semi-structured interview (Principals, administrators, teachers, recess coach),  Semi-structured playground observation | Coding system consistent with Grounded theory approach | Data source triangulation,  Investigator triangulation, inter-rater reliability |
| London 2022 [11],  **USA *** | Child development and play | To explore how to organise breaktime to best support the developmental needs of middle schoolers. | Exploratory design | Schools: Purposive, allowed year-round outdoor recess  Participants: NR | Middle (n=**3**),  50% students eligible for free lunches. | Middle school staff (n=**14**)  Elementary school Principals (n=**4**)  School District personnel (n=**2**) | Semi-structured interview,  Playground observation,  Field notes | Emergent coding of interview data to draw out themes | Collaborative review of field notes |
| McNamara et al. 2013 [12],  **Canada ^ⴕ^** | Child development and play | To highlight children’s voices about their recess environment, and add perspectives from the teachers, principals, and playground volunteers. | Inductive qualitative approach | Schools: Purposive sampling based on risk factors for low education outcomes.  Participants: Purposive (maximum variation sampling) to gain a range of perspectives. | Elementary (n=**4**),  South Ontario,  Low SES. | Teachers (n=**22**).  Principals/ Administrators (n=**4**), Playground Volunteers (n=**9**).  Yard duty supervisors (n=**3**). | Questionnaires, with open-ended questions,  Observations,  Field notes,  Journals,  Interviews, unstructured | Data coded inductively; themes developed | Member checking, data source triangulation, method triangulation |
| Niehues et al. 2013 [13],  **Australia** | Psychology | To investigate and alter adults’ perceptions of risk to increase the sustainability of a child-centred playground intervention | Theories of flourishing,  positivity and SDT | Schools: Purposive (based on participant SES, culture, mothers’ education). Part of larger SPP.  Participants: Convenience sampling (parents and teachers of children from SPP schools) | Elementary (n=**9**), urban, Sydney,  Catholic schools, Low & Med SES. | Parents (n=~**103**),  Teachers (n=~**35**),  Other school staff (n=**12**),  majority female. | Audio recorded workshops (risk-reframing sessions),  Field notes,  Participant evaluation surveys | Adaption of Charmaz's approach to social analysis, constant comparative analysis. | Investigator triangulation, collaborative review of themes |
| Niehues et al. 2016 [14],  **Australia** | Psychology | To investigate the dilemmas adults experience in balancing protecting children and offering age-appropriate risk-taking opportunities. | Broaden and Build  Theory of Positive Emotions, Theories of risk,  play motivation,  resilience | Schools: Recruited as part of larger SPP.  Participants: Convenience (participated in previous risk-reframing workshops and indicated interest to participate in further research). | Elementary (n=**NR**), urban, Sydney,  Catholic schools. | Parents (n=**37**).  Teacher (n=**8**). | Interviews, semi-structured and in depth | Hermeneutic interpretive analysis, combining narrative, thematic and theoretical lenses. | Data source triangulation, theory triangulation |
| Norodahl et al. 2015 [15],  **Iceland ^ⴕ^** | Environmental education and health | To explore children’s preferences about outdoor activities and surroundings in the outdoor school environment. | Participatory research design to inform school grounds project,  Theory of affordances | Schools: Recruited as part of a larger project on Education for sustainable development.  Participants: students selected by teachers using maximum variation approach (gender, age, neighborhoods, interests). | Elementary (n=**1**),  and preschool (n=**2**), urban, small municipality near Reykjavík. | Teachers (n=**15**).  Principals/ Administrators (n=**3**). | Interviews, individual and group,  Observations,  Field notes,  Meetings | Thematic analysis, using Braun & Clarke’s 6-step process | Data source triangulation, method triangulation |
| Ozkal et al. 2020 [16],  **Turkey** | Child development and play | To determine the opinions of the school administrators and teachers working in primary and secondary schools on the role of recess for students. | Qualitative descriptive design,  Phenomenology | Schools: NR  Participants: Purposive, maximum variation sampling method (school, gender, branch, and seniority). | Elementary (n=**2**), Secondary (n=**2**),  Med SES. | Teachers (n=**20**),  60% female. Principals/ Administrators (n=**11**), 100% male. | Interviews, semi-structured | Content analysis, using deductive and inductive coding. | Member checking, peer debrief, critical friends |
| Parrish et al. 2012 [17],  **Australia ^ⴕ^** | Public health: PA promotion | To understand how physical and psychosocial school environmental variables influence children’s playground PA levels. | Qualitative descriptive design,  SCT | Schools: Extreme case sampling method (least and highest active schools in larger study).  Participants: Children from Grades 4-6 self-selected; One administrator from each school; Teachers NR. | Elementary (n=**6**),  Low & Med SES, High and low PA schools. | Teachers (n=**16**), 100% female.  Principals/ Administrators (n=**6**), 50% female. | Interviews, semi-structured | Data coded inductively; themes developed. | Data source triangulation, method triangulation (from the larger study). |
| Peterson et al. 2018 [18],  **Canada** | Child development and play | To explore teachers perspectives on the place of rough and tumble play, and teachers’ roles vis-à-vis rough and tumble play in schools | Collaborative action research | Schools: Recruited as part of larger (NOW Play) project.  Participants: Purposive - Participants were part of the NOW Play project. | Elementary (n=**3**), small northern rural and Indigenous communities. | Teachers (n=**8**),  88% female.  Indigenous consultant teachers (n=**2**), 100% female. | FG, semi-structured  Video-elicitation | Framework analysis, using inductive and deductive coding | Member checking |
| Ren et al. 2010 [19],  **USA ^ⴕ^** | Psychology | To work with children to assess and improve the institutional setting of recess. | Participatory action research design, Collaborative  community psychology | Schools: Invitation by Principal to help reform the playground.  Participants: Theoretical sampling, extreme and normative case sampling. | Elementary (n=**1**). | Recess aides (n=**4**) | FG, semi-structured  Observations | Data coded inductively; themes developed | Data source triangulation, investigator triangulation, inter-rater reliability |
| Sharkey et al. 2014 [20],  **USA ^ⴕ^** | Education | To conduct a needs assessment and develop a customized training for yard supervisors at a public elementary school to help reduce student conflicts and promote a positive school climate. | Formative evaluation (Needs Assessment) | Schools: NR  Participants: NR | Elementary (n=**1**),  Central California,  Low SES. | Yard duty supervisors (n=**11**) | Interviews, semi-structured | Data coded inductively; themes developed | Peer debrief, collaborative review of themes |
| Stevens et al. 2020 [21],  **Canada** | Environmental education and health | To develop an evaluation of the NPLS program to capture the stories of parents, teachers, and  administrators who were involved with the program. | Case study, Collaborative participatory approach; narrative program evaluation, program theory | Schools: Recruited as part of Nature Play and Learning Spaces (NPLS) program.  Participants: NR, adults who participated in the program. | Elementary (n=**5**). | Students (n=**NR**)  Teachers (n=**6**),  Principals/ Administrators (n=**6**),  Parents (n=**3**),  Program mentor (n=**1**) | Interviews, semi-structured & conversational,  Observations in playgrounds | Framework analysis,  narrative analysis from a constructionist perspective,  logic modelling | Member checking, data source triangulation |
| Suleman et al. 2021 [22],  **USA *** | Public health: PA promotion | To examine the implementation of the CDC strategy in an urban, inner-city elementary school to identify barriers and facilitators to successful recess implementation from the perspective of various stakeholders. | Formative research study design  Phenomenology | Schools: NR  Participants: Purposive & snowballing (parents) | Elementary (n=**1**), urban, inner city,  99% students black, 87% eligible for free/ reduced lunches. | Teachers (n=**5**),  Parents (n=**3**),  Recess monitors (n=**12**),  Principals/ Administrators (n=**3**),  87% female, 9% male, <4% non-binary | In-depth interviews (teachers, principals, administrators),  FG (parents, recess monitors),  Unstructured playground observations, field notes | Deductive content analysis | Data source triangulation, Peer debriefing, collaborative review of themes |
| Thomson et al. 2003 [23],  **England ^ⴕ^** | Child development and play | To explore the nature of contemporary primary school playground activities and whether these activities reflect the definition of play. | NR | Schools: NR  Participants: NR | Elementary (n=**4**), urban, suburban, and rural,  Variety of SES. | Principals/ Administrators (n=NR),  Teachers (n=NR),  Midday assistants (n=NR). | Observation in playground,  Interviews, informal | NR | NR |
| Thomson et al. 2005 [24],  **England ^ⴕ^** | Human geography | To examine the spatial strategies used by playground supervisors, and present children’s territorialization of the playground and  responses to adults’ spatial control. | Ethnography,  Theory of human territoriality | Schools: Stratified random selection based on urban, suburban and rural location within a defined geographical area.  Participants: NR | Elementary (n=**3**), 1 each urban, suburban, and rural, Northern England & Midlands,  Low – Med SES. | Principals/ Administrators (n=**3**),  Teachers (n=NR),  Midday assistants (n=NR). | Ethnographic and  Systematic observation using RPI tool,  Field notes, Photographs,  Interviews, semi-structured and informal | Ethnographic analysis | Immersion in research context across three years |
| Thomson et al. 2007 [25],  **England ^ⴕ^** | Education | To elucidate children’s experience of the school playground environment and explore how adults limit children's interaction with the environment. | Ethnography,  Free choice learning | Schools: Stratified random selection based on urban, suburban and rural location within a defined geographical area.  Participants: NR | Elementary (n=**3**), 1 each urban, suburban, and rural, Northern England & Midlands,  Low – Med SES. | Principals/ Administrators (n=NR),  Teachers (n=NR),  Playtime supervisors (n=NR). | Ethnographic and  Systematic observation using RPI tool,  Field notes,  Photographs,  Interviews, semi-structured and informal | Ethnographic analysis | Immersion in research context across three years |

Legend: CDC = Centres for Disease Control; FG = Focus Group; Med = Medium; N/A = Not Applicable; NASN = National Association of School Nurses; NR = Not Reported; NOW Play = Northern Oral language and Writing through Play; NPLS = Nature Play and Learning Spaces; NW = North-western; PA = Physical Activity; RCT = Randomised Controlled Trial; RPI = Record of Perceived Intervention; SCT = Social Cognitive Theory; SDT = Social Determination Theory; SEM = Social Ecological Model; SES = Socio-Economic Status; SPP = Sydney Playground Project.

**^ⴕ^** Studies involved both adult and child participants, however, only adult population and methods described.

* Studies added in updated search June 2022.

# References

1. Bundy, A.C., et al., *The risk is that there is ‘no risk’: a simple, innovative intervention to increase children’s activity levels.* International Journal of Early Years Education, 2009. **17**(1): p. 33-45.

2. Farmer, V.L., et al., *What did schools experience from participating in a randomised controlled study (PLAY) that prioritised risk and challenge in active play for children while at school?* Journal of Adventure Education and Outdoor Learning, 2017. **17**(3): p. 239-257.

3. Graham, M., et al., *A socio-ecological examination of the primary school playground: Primary school pupil and staff perceived barriers and facilitators to a physically active playground during break and lunch-times.* PLoS ONE, 2022. **17**(2): p. e0261812.

4. Gyllencreutz, L., et al., *Injury risks during outdoor play among Swedish schoolchildren: teachers’ perceptions and injury preventive practices.* Education 3-13, 2020. **48**(1): p. 1-11.

5. Harper, N.J., et al., *A case study exploring the ‘real world’ process of ‘naturalizing’ school playgrounds.* International Journal of Environmental Health Research, 2019.

6. Hudson, S.D., H.M. Olsen, and D. Thompson, *An Investigation of School Playground Safety Practices as Reported by School Nurses.* Journal of School Nursing, 2008. **24**(3): p. 138-144.

7. Jarvis, P., *Dangerous Activities within an Invisible Playground: A Study of Emergent Male Football Play and Teachers' Perspectives of Outdoor Free Play in the Early Years of Primary School.* International Journal of Early Years Education, 2007. **15**(3): p. 245-259.

8. Kuru, G., E.D. Öztürk, and F. Atmaca, *A Field of learning and living: Suitability of school gardens for children.* Ilkogretim Online, 2020. **19**(3): p. 1450-1464.

9. Larsson, A. and M. Rönnlund, *The spatial practice of the schoolyard. A comparison between Swedish and French teachers' and principals' perceptions of educational outdoor spaces.* Journal of Adventure Education and Outdoor Learning, 2021. **21**(2): p. 139-150.

10. London, R.A., et al., *Playing fair: the contribution of high-functioning recess to overall school climate in low-income elementary schools.* J Sch Health, 2015. **85**(1): p. 53-60.

11. London, R.A., *It is not called recess anymore: Breaktime in middle school.* The Journal of School Health, 2022. **Epub ahead of print**.

12. McNamara, L., *What's Getting in the Way of Play? An Analysis of the Contextual Factors that Hinder Recess in Elementary Schools.* Canadian Journal of Action Research, 2013. **14**(2): p. 3-21.

13. Niehues, A.N., et al., *Everyday uncertainties: reframing perceptions of risk in outdoor free play.* Journal of Adventure Education and Outdoor Learning, 2013. **13**(3): p. 223-237.

14. Niehues, A.N., et al., *Reframing healthy risk taking: Parents’ dilemmas and strategies to promote children’s well-being.* Journal of Occupational Science, 2016. **23**(4): p. 449-463.

15. Norðdahl, K. and J. Einarsdóttir, *Children's Views and Preferences Regarding Their Outdoor Environment.* Journal of Adventure Education and Outdoor Learning, 2015. **15**(2): p. 152-167.

16. Özkal, N., *Teachers' and School Administrators' Views Regarding the Role of Recess for Students.* International Journal of Progressive Education, 2020. **16**(5): p. 121-137.

17. Parrish, A.M., et al., *Using interviews and peer pairs to better understand how school environments affect young children's playground physical activity levels: a qualitative study.* Health Education Research, 2012. **27**(2): p. 269-280.

18. Peterson, S.S., et al., *Children's Rough and Tumble Play: Perspectives of Teachers in Northern Canadian Indigenous Communities.* Early Years: An International Journal of Research and Development, 2018. **38**(1): p. 53-67.

19. Ren, J.Y. and R.D. Langhout, *A recess evaluation with the players: taking steps toward participatory action research.* American journal of community psychology, 2010. **46**(1-2): p. 124-138.

20. Sharkey, J.D., et al., *Effective yard supervision: From needs assessment to customized training.* Contemporary School Psychology, 2014. **18**(2): p. 103-116.

21. Stevens, Z., et al., *Shifting Culture Towards Endorsement and Advocacy of Outdoor Play and Learning: A Collaborative Case Study with KidActive.* Canadian Journal of Environmental Education, 2020. **23**(2): p. 106-124.

22. Suleman, S., et al., *Implementation of CDC guidelines for recess: A formative research study.* Health Promotion Practice, 2021. **Epub ahead of print**.

23. Thomson, S., *A well-equipped hamster cage: The rationalisation of primary school playtime.* Education 3-13, 2003. **31**(2): p. 54-59.

24. Thomson, S., *‘Territorialising’ the primary school playground: deconstructing the geography of playtime.* Children's Geographies, 2005. **3**(1): p. 63-78.

25. Thomson, S., *Do’s and don’ts: children’s experiences of the primary school playground.* Environmental Education Research, 2007. **13**(4): p. 487-500.
