# Supplementary material for: “Children are precious cargo; we don’t let them take any risks!”: Hearing from adults on safety and risk in children’s active play in schools: a systematic review
Source: Int J Behav Nutr Phys Act. 2022 Sep 1;19:111. doi: 10.1186/s12966-022-01344-7 (PMC9438168; doi:10.1186/s12966-022-01344-7)
Supplement: Supplementary file 5 — Additional file 5. Quality appraisal of included studies. Description: Appraisal results table for all studies using the CASP Qualitive Checklist. [file 12966_2022_1344_MOESM5_ESM.docx]

# Additional file 5: Quality Appraisal of included studies

|  | **SCREENING QUESTIONS** | | **APPRAISAL QUESTIONS** | | | | | | | |
| --- | --- | --- | --- | --- | --- | --- | --- | --- | --- | --- |
| **CASP CHECKLIST** | **1. Aims of study** | **2. Appropriateness of qualitative methodology** | **3. Research design** | **4. Recruitment strategy** | **5. Data collection** | **6. Reflexivity-related issues** | **7. Ethical issues** | **8. Rigor of data analysis** | **9. Reporting of study findings** | **10. Value of study findings** |
| Bundy *et al.* 2009 | 1 | 1 | 1 | 0.5 | 1 | 0.5 | 0.5 | 0.5 | 1 | 1 |
| Farmer *et al.* 2017 | 1 | 1 | 1 | 0 | 1 | 0 | 1 | 1 | 1 | 1 |
| Graham *et al.* 2022 | 1 | 1 | 1 | 1 | 1 | 0.5 | 1 | 1 | 1 | 1 |
| Gyllencreutz *et al.* 2020 | 1 | 1 | 1 | 0.5 | 1 | 0 | 1 | 1 | 0.5 | 1 |
| Harper *et al.* 2019 | 1 | 1 | 1 | 1 | 1 | 0 | 0.5 | 1 | 1 | 1 |
| Hudson *et al.* 2008 | 1 | 1 | 0.5 | 1 | 1 | 0 | 0.5 | 0.5 | 1 | 1 |
| Jarvis *et al.* 2007 | 0.5 | 1 | 1 | 0 | 1 | 1 | 0 | 1 | 1 | 1 |
| Kuru *et al.* 2020 | 1 | 1 | 1 | 1 | 0.5 | 0 | 0 | 1 | 1 | 0.5 |
| Larsson *et al.* 2021 | 1 | 1 | 1 | 0.5 | 0.5 | 0.5 | 1 | 0 | 0.5 | 0.5 |
| London *et al.* 2015 | 1 | 1 | 0.5 | 0.5 | 0.5 | 0 | 1 | 0.5 | 1 | 1 |
| London 2022 | 1 | 1 | 0.5 | 0.5 | 0.5 | 0 | 0.5 | 0.5 | 0.5 | 0.5 |
| McNamara *et al.* 2013 | 1 | 1 | 1 | 1 | 1 | 0.5 | 0 | 1 | 1 | 1 |
| Niehues *et al.* 2013 | 1 | 1 | 1 | 1 | 0.5 | 0 | 0.5 | 1 | 1 | 1 |
| Niehues *et al.* 2016 | 1 | 1 | 1 | 1 | 1 | 0 | 1 | 1 | 1 | 1 |
| Norodahl *et al.* 2015 | 1 | 1 | 1 | 1 | 1 | 0 | 1 | 1 | 1 | 1 |
| Ozkal *et al.* 2020 | 1 | 1 | 1 | 1 | 1 | 0 | 0 | 1 | 1 | 1 |
| Parrish *et al.* 2012 | 1 | 1 | 1 | 1 | 1 | 0 | 1 | 0.5 | 1 | 1 |
| Peterson *et al.* 2018 | 1 | 1 | 1 | 1 | 1 | 0 | 0 | 1 | 1 | 1 |
| Ren *et al.* 2010 | 1 | 1 | 1 | 1 | 1 | 0 | 1 | 1 | 1 | 1 |
| Sharkey *et al.* 2014 | 1 | 1 | 1 | 0 | 1 | 0 | 0.5 | 1 | 1 | 1 |
| Stevens *et al.* 2020 | 1 | 1 | 1 | 1 | 1 | 0 | 1 | 1 | 1 | 1 |
| Suleman *et al.* 2021 | 1 | 1 | 1 | 0.5 | 0.5 | 0 | 1 | 1 | 1 | 1 |
| Thomson *et al.* 2003 | 0.5 | 0 | 0.5 | 0 | 0 | 0 | 0 | 0 | 0.5 | 0.5 |
| Thomson *et al.* 2005 | 1 | 1 | 0.5 | 1 | 1 | 0 | 1 | 1 | 0.5 | 1 |
| Thomson *et al.* 2007 | 1 | 1 | 1 | 1 | 1 | 1 | 1 | 0.5 | 1 | 1 |

Legend: Totally met = 1; Somewhat met = 0.5; Not met = 0; Can’t tell = 0
